# Antineoplastic Effects of siRNA against TMPRSS2-ERG Junction Oncogene in Prostate Cancer

**DOI:** 10.1371/journal.pone.0125277

**Published:** 2015-05-01

**Authors:** Giorgia Urbinati, Hafiz Muhammad Ali, Quentin Rousseau, Hubert Chapuis, Didier Desmaële, Patrick Couvreur, Liliane Massaad-Massade

**Affiliations:** 1 Université Paris-Sud 11, Laboratoire de Vectorologie et Thérapeutiques Anticancéreuses, UMR 8203, Villejuif, France-94805; 2 CNRS, Villejuif, Laboratoire de Vectorologie et Thérapeutiques Anticancéreuses, UMR 8203, Villejuif, France-94805; 3 Gustave Roussy, Laboratoire de Vectorologie et Thérapeutiques Anticancéreuses, UMR 8203, Villejuif, France-94805; 4 Laboratory of Experimental Cancer Research, Ghent University Hospital Building P7, De Pintelaan 185, B-9000 Gent, Belgium; 5 Institut Galien, UMR CNRS 8612, Université Paris-Sud 11, Faculté de pharmacie, 5 rue J. B. Clément, 92296 Châtenay-Malabry, France; University of Kentucky College of Medicine, UNITED STATES

## Abstract

*TMPRSS2-ERG* junction oncogene is present in more than 50% of patients with prostate cancer and its expression is frequently associated with poor prognosis. Our aim is to achieve gene knockdown by siRNA TMPRSS2-ERG and then to assess the biological consequences of this inhibition. First, we designed siRNAs against the two *TMPRSS2-ERG* fusion variants (III and IV), most frequently identified in patients’ biopsies. Two of the five siRNAs tested were found to efficiently inhibit mRNA of both *TMPRSS2-ERG* variants and to decrease ERG protein expression. Microarray analysis further confirmed ERG inhibition by both siRNAs TMPRSS2-ERG and revealed one common down-regulated gene, *ADRA2A*, involved in cell proliferation and migration. The siRNA against TMPRSS2-ERG fusion variant IV showed the highest anti-proliferative effects: Significantly decreased cell viability, increased cleaved caspase-3 and inhibited a cluster of anti-apoptotic proteins. To propose a concrete therapeutic approach, siRNA TMPRSS2-ERG IV was conjugated to squalene, which can self-organize as nanoparticles in water. The nanoparticles of siRNA TMPRSS2-ERG-squalene injected intravenously in SCID mice reduced growth of VCaP xenografted tumours, inhibited oncoprotein expression and partially restored differentiation (decrease in Ki67). In conclusion, this study offers a new prospect of treatment for prostate cancer based on siRNA-squalene nanoparticles targeting *TMPRSS2-ERG* junction oncogene.

## Introduction

Prostate cancer (PCa), an androgen-dependent tumour, has become the most frequent cancer in men (27% of all cancers in men) and represents the 4^th^ cause of mortality by cancer and the 2^nd^ in men. In 2014, the estimated incidence was of approximately 230,000 cases in the United States and 417,000 cases in Europe (ACS. American Cancer Society, *acs*.*org*; WHO. European Cancer Observatory, *eu-cancer*.*iarc*.*fr*). The main risk factors include age, family history and black ethnic origin but carcinogenesis results from an interaction between both environmental and endogenous factors [1]. The conventional treatment for prostate cancer is androgen deprivation therapy; however, despite a high response to this therapy, most patients progress to castration resistance [2]. Since 2010, five new US Food and Drug Administration-approved treatments have been available for castration-resistant and metastatic prostate cancers (Cabazitaxel, Enzalutamide, Abiraterone acetate, Sipuleucel T, ^223^Radium dichloride). However, the side effects of these new drugs are not yet well-established and patient survival is only slightly improved, resulting in palliative rather than therapeutic treatments [1, 3]. Consequently, the development of new therapies is still highly recommended and indispensable.

Tomlins *et al*., discovered a gene fusion named TMPRSS2-ERG in more than 50% of prostate cancers [4]. TMPRSS2-ERG is due to rearrangement affecting chromosome 21 leading to the fusion of a gene regulated by androgens *TMPRSS2*, with the transcriptional factor *ERG* [5]. The fusion of *TMPRSS2-ERG* leads to over-expression of ERG in the prostate gland; this promotes prostate tumour initiation and progression. Consistently, a significant amount of data suggest that this fusion gives a more aggressive phenotype and may affects the outcome of localized tumours treated with androgen deprivation therapy [5–11]. More than 17 transcripts have been observed for *TMPRSS2-ERG* junction oncogene and the best known, described by Wang *et al*. are designated as variants I to VIII [12]. Amongst them, the most frequent variants found in patients’ biopsies are variants III and IV and result from joining exon 1 of *TMPRSS2* with exons 4 or 5 of *ERG*, respectively. Both fusions lead to an over-expression of truncated but functional ERG protein [12–14]. Moreover, various forms of fusion transcripts were described within the same tumour [15, 16].

Despite the fact that small interfering RNAs (siRNAs) are highly specific and efficient at very low concentrations, they are unstable in biological fluids and the hydrophilic character hinders their target delivery and cellular uptake. To improve their stability, protect them from nucleases degradation and enhance their hydrophobic character, they need to be better transported and delivered into the body [17]. We recently proved the efficiency of the “squalenoylation” technology to vectorise siRNAs designed against *RET/PTC1* and *RET/PTC3* junction oncogenes, and suggested that squalenoylation offers a new non-cationic platform for siRNA delivery [18, 19]. Knowing that a significant percentage of prostate malignancy harbours the *TMPRSS2-ERG* junction oncogene, our aim is to introduce a new potential therapeutic approach by siRNA targeting *TMPRSS2-ERG* junction oncogene in patients with prostate cancer. Our results point out a concrete clinical application for prostate cancer therapy based on TMPRSS2-ERG knockdown.

## Material and Methods

### Chemicals

All the chemicals used were of highest analytical grade. Squalene, siRNAs, MTT [3-(4,5-dimethylthiazol-2-yl)-2,5-diphenyl tetrazolium bromide] reagent and paraformaldehyde (PFA, 16%) were purchased from Sigma-Aldrich Chemical Co. (Saint Quentin Fallavier, France). 3’-thiol modified siRNAs were purchased from Eurogentec (Belgium) and Dulbecco’s modified Eagle medium (DMEM), Opti-MEM, fetal calf serums (FCS), Lipofectamine RNAiMAX and PCR primers were purchased from Life Technologies (Saint Aubin, France). BD Matrigel (Basement Membrane Matrix Growth Factor Reduced—Reference 356234) was purchased from Corning (Amsterdam, the Netherlands). Bio-RAD protein assay was purchased from Bio-RAD Laboratories (Marnes-la-Coquette, France). Annexin-V-Fluos staining kit was purchased from Roche (Meylan, France). NucView 488 caspase-3 kit was purchased from VWR (Fontenay-sous-Bois, France). Proteome Profiler Human Apoptosis Array kit was purchased from R&D Systems (Lille, France). Fluoromount-G was purchased from Clinisciences (Nanterre, France). Water was purified using a Milli-Q system (Millipore, Saint Quentin en Yvelines, France).

### Cell lines and cell culture

Human prostate cancer VCaP cell line expressing *TMPRSS2-ERG* oncogene (ATCC CRL-2876 Manassas, USA) was grown in Dulbecco's Modified Eagle Medium (DMEM) supplemented with FCS, 100 units/ml penicillin and 100 μg/ml streptomycin (Invitrogen, Cergy-Pontoise, France). Cells were incubated at 37°C in a humidified atmosphere containing 5% CO_2_. Before the beginning of experiments, the cells were analysed by polymerase chain reaction (PCR) and were found to be free from mycoplasma.

### Oligonucleotides design and determination of *TMPRSS2-ERG* variants in VCaP cells

In order to detect the TMPRSS2-ERG variants in VCaP cells, 10 sets of primers were designed either within the *TMPRSS2* or *ERG* genes or across both genes for variants I to VIII of *TMPRSS2-ERG* (S1 Table). Amplifications were performed by reverse transcription (RT) followed by real time quantitative PCR (qPCR).

### siRNAs design against TMPRSS2-ERG variants III and IV

The TMPRSS2-ERG mRNA sequence was obtained by blasting TMPRSS2-ERG with Human TMPRSS2 mRNA (NM: 005656.2) and Homo sapiens ERG mRNA sequence (NM: 004449.3). We designed five siRNAs according to Reynolds’ rules [20] against the most frequent and abundant TMPRSS2-ERG fusion variants found in patients and VCaP cells. Three siRNAs were designed for variant III, named siRNA TMPRSS2-ERG III (1), III (2), III (3), and two siRNAs against TMPRSS2-ERG fusion variant IV, named siRNA TMPRSS2-ERG IV (1) and IV (2); their sequences are enlisted in S2 Table. The siRNA control has the sequence of the siRNA TMPRSS2-ERG IV (1) with five mismatches. All single-stranded siRNAs were synthesized by Sigma-Aldrich Chemical Co. (Saint Quentin Fallavier, France) as 21-mer with two 3’-overhanging 2’-deoxynucleotide residues to provide stabilization against nucleases [21]. In order to perform squalene bio-conjugation, a 3-mercaptopropyl phosphate group was introduced at the 3'-end of siRNA sense strand (synthetized by Eurogentec, Belgium).

### *In vitro* cell transfection

Transient transfections were performed in order to: i) assess the most efficient siRNA TMPRSS2-ERG designed [siRNA III (1, 2, 3), or IV (1, 2)], ii) find the most efficient siRNA concentration, iii) assess the efficiency of siRNAs, iv) analyse the knockdown effects on cell viability, apoptosis and gene regulation, v) verify the *TMPRSS2-ERG* knockdown efficiency, with and without transfecting agents, after siRNA TMPRSS2-ERG squalenoylation.

Transient transfections were carried out using Lipofectamine RNAiMAX transfecting agent according to manufacturer's instructions. Briefly, 8×10^5^ VCaP cells were seeded in six-well plates containing DMEM supplemented with 10% FCS, penicillin (100U/ml) and streptomycin (10μg/ml) using different siRNA concentrations and 6 μL Lipofectamine RNAiMAX. Cells were incubated with siRNA for 24h, 48h and 72h. FAM-labeled siRNA Control was used to monitor the efficiency of siRNA transfection.

For the choice of an efficient siRNA concentration, VCaP cells were transfected with the selected efficient siRNAs TMPRSS2-ERG III or IV at 2.5 nM, 10 nM, 25 nM and 50 nM concentrations and incubated for 48h. At the end of the treatments, mRNA and proteins were extracted from the cells to be analysed for gene and protein knockdown.

### Real time PCR (RT-qPCR)

Total RNA was extracted from VCaP cells using RNeasy mini-kit (Qiagen, Courtaboeuf, France). First-strand cDNA was generated with M-MLV RT buffer pack (Promega, Charbonnières-les-Bains, France). Real-time PCR (qPCR) was carried out with StepOnePlus PCR System (AB Applied Biosystems, Villebon-sur-Yvette, France) using GoTaq qPCR Master Mix (Promega, Charbonnières-les-Bains, France) according to manufacturer’s instructions. Samples were run in triplicate; gene regulation was determined by 2^-ΔΔCt^ method and normalized to GAPDH levels. For knockdown experiments, results are given as relative mRNA levels compared to non-treated cells.

### Immunoblotting

Total protein extracts were obtained using M-PER reagent (Thermo Fisher Scientific Courtaboeuf, France) supplemented with a protease inhibitor cocktail (Roche, Neuilly sur Seine, France). Proteins were titrated by Bio-RAD Assay according to manufacturer’s instructions. Samples were then loaded on 10% polyacrylamide gel (NuPAGE Bis Tris Mini Gels 10%, Life technologies, Saint-Aubin, France) and proteins were transferred using the iBlotDry Blotting System (Invitrogen, France). Membranes were incubated overnight at 4°C with either of the following primary antibodies: monoclonal rabbit ERG (EPR 3864 (2); 1:500, Abcam Biochemicals, Paris, France) or monoclonal rabbit Caspase-3 (1:1000, Cell Signalling technology, Saint Quentin en Yvelines, France. Ref: 9662). Monoclonal mouse GAPDH-HRP (1:1000, Cell Signalling technology, Saint Quentin en Yvelines, France. Ref: 3683) was used as internal control. Blots were then washed and incubated with corresponding secondary anti-rabbit or anti-mouse antibodies conjugated to HRP (horseradish peroxidase, 1:3000, Cell Signalling technology). Bands were visualized by enhanced chemiluminescence reagent (Invitrogen, France).

### Viability assay

The viability of VCaP cells was evaluated by MTT assay after 72h incubation with siRNAs TMPRSS2-ERG or Control at 50 nM concentration. MTT assay was also performed 72h after cell transfection with siRNA TMPRSS2-ERG III and IV at 0.1 nM, 1 nM, 2.5 nM, 10 nM, 25 nM, 50 nM, 100 nM, 150 nM and 200 nM concentrations. Results are the mean ± SD of two independent experiments containing 8 replicates for each condition and are expressed as viability percentage of treated cells compared to non-treated cells.

### Whole-genome microarray analysis

Three independent transfections were performed with 50 nM siRNA TMPRSS2-ERG III, siRNA TMPRSS2-ERG IV and siRNA Control on VCaP cells. Total RNAs of untreated and transfected cells were extracted using RNeasy mini-kit (Qiagen, Courtaboeuf, France). Protocol is detailed in Array Express (Accession Number: E-MTAB-2838) and previously described by Gilbert-Sirieix [22]. The mRNA was then labelled using fluorescent low-input linear amplification kit (Agilent Technologies, Massy, France). Briefly, reverse transcription was performed using M-MLV reverse transcriptase. Then, cyanine 3-labelled cDNAs were generated using T7 RNA polymerase. Hybridizations were carried out for 17h at 60°C on Agilent human whole genome oligo microarray 8x60k, either with 1 μg of untreated cells or with siRNA TMPRSS2-ERG III, siRNA TMPRSS2-ERG IV or siRNA Control transfected cells. Slides were scanned using Agilent 2505 C DNA microarray scanner and microarray images were analysed with Feature extraction software version 10.7.3.1 (Agilent Technologies). Raw data files were normalized with Limma procedure. We defined up- or down-regulations as ratios greater than two-fold between VCaP cells transfected with siRNA (TMPRSS2-ERG III, TMPRSS2-ERG IV and Control sequences) and untreated VCaP cells, acquired with a fold discovery rate ≤ 0.05 and a minimum intensity ≥ 100. In order to interpret the biological meaning of the genomic data, we used the ingenuity software (www.ingenuity.com). Then, the authenticity of microarray data was validated by RT-qPCR, of few of the TOP 10 up- or down-regulated genes by siRNA TMPRSS2-ERG III and IV. Gene-specific primers were designed using the Oligo Explorer 1.1.0 and Oligo Analyzer 1.0.2 programs (Kuopio University, Kuopio, Finland, primers sequences are enlisted in S3 Table). Primers were selected with a melting temperature of 60°C and an amplicon size of 100–200 bases. Samples were run in duplicate with primer sets of the gene of interest and the *GAPDH* as internal control gene.

### Annexin-V apoptosis assay

Apoptosis was determined by flow cytometry analysis using an Annexin-V-Fluos staining kit containing both Annexin-V bound to fluorescein and propidium iodide (PI). VCaP cells were transfected with 50 nM of siRNA TMPRSS2-ERG III, IV or siRNA Control in the presence of Lipofectamine RNAiMAX. After 72h or 96h incubations, medium and cells were collected and centrifuged, then stained using the Annexin-V-Fluos staining kit according to manufacturer’s instructions. The samples were then analysed by flow cytometry (Accuri C6 Flow Cytometer, BD Biosciences, San Jose, USA). Ten thousand events in the selected population were analysed and electronic compensation of the instrument was performed to exclude overlapping of the two emission spectra (Fluorescein and PI). Experiments were performed in triplicate and treated cells in each phase of cell death were compared to non-treated cells.

### Cell apoptosis assays

Apoptosis was evaluated by IncuCyte system (Essen Instruments, Ann Arbor, Michigan, USA), which allows monitoring of living cells in culture over time and quantifies apoptotic cells by counting fluorescent nuclei present per mm^2^ area in every image. Caspase-3/7 substrate (NucView488 caspase-3 kit) was used to follow caspase activity in living cells in real-time. When the substrate is cleaved by activated caspase-3/7, the high-affinity DNA dye is released and migrates to the cell nucleus to stain it brightly. Wells were scanned (6 images/well) every 12h by IncuCyte for monitoring of fluorescence for each image.

VCaP cells (30,000/well) were seeded in 96 well plates with 100 μL of DMEM containing FCS and antibiotics, and transfected with 50 nM of siRNA TMPRSS2-ERG III, IV or siRNA Control. Apoptosis detection kit NucView 488 caspase-3/7 was added and cell plates were placed in the IncuCyte under incubation conditions. The experiment was performed in triplicate and the fluorescent apoptotic signal in siRNA transfected cells was compared to that of non-treated cells.

### Proteome apoptosis profiler array

First, transient transfection of siRNAs TMPRSS2-ERG III, IV and siRNA Control was carried out in VCaP cells for 72 hours, as described above. Cells were lysed and protein concentration was determined by Bio-RAD assay according to manufacturer’s instructions. Each protein sample (non-treated VCaP cells, treated VCaP cells with siRNAs TMPRSS2-ERG III, IV or siRNA Control) was then incubated overnight with the “Human Apoptosis Array” (R&D) (300 μg of proteins/array), a nitrocellulose membrane having target and control antibodies spotted in duplicate. After incubation of samples with nitrocellulose membranes, arrays were washed to remove unbound proteins and then incubated with a cocktail of biotinylated detection antibodies. Membranes were then incubated with streptavidin-HRP reagent, washed again and revealed with the chemiluminescent detection reagent. The intensity of dots corresponds to the amount of protein bound to the spotted antibodies.

### Preparation and *in vitro* cellular uptake of siRNA TMPRSS2-ERG-squalene nanoparticles

The bio-conjugate siRNA TMPRSS2-ERG-squalene (SQ) was synthesized by Michael addition of 3’-thiol group with squalene-(ethoxy)ethyl-maleimide and the corresponding nanoparticles (NPs) were prepared by nano-precipitation as previously published [23]. The hydrodynamic diameter (nm) and the Zeta potential (mV) were measured by laser light scattering using a Zetasizer 4 (Malvern Instrument Ltd, Orsay, France).

The cellular uptake of siRNA TMPRSS2-ERG-SQ nanoparticles was then investigated. One day prior to transfection, VCaP cells were seeded at 8×10^5^ cells/well into 6-well plates containing a cover glass. Vectorized FAM-labeled siRNA-SQ NPs (50 nM) were directly added to VCaP cells or transfected with Lipofectamine RNAiMAX transfecting agent, as previously described. After 24h incubation at 37°C, medium was removed and cells were washed twice with PBS then fixed with 4% formaldehyde for 20 min. After final rinses in PBS, nuclei were stained with Fluoromount-G mounting medium containing 4’,6-diamidino-2-phenylindole (DAPI) and labeled cells were detected by fluorescence microscopy (Zeiss LSM510/Axiovert 200M, Carl Zeiss SAS, Marly le Roi, France).

### Animal studies

All animal experiments and the use of VCaP cells were approved by the institutional Ethics Committee of Animal Experimentation (CEEA) and research council (Integrated Research Cancer Institute in Villejuif, IRCIV), registered in the French Ministry of Higher Education and Research (Ministère de l’Enseignement Supérieur et de la Recherche; MESR) under the authorization number CEEA IRCIV/IGR n°26: 94–226, n°: 2011–09 and carried out according to French laws and regulations under the conditions established by the European Community (Directive 2010/63/UE). All efforts were made to minimize animal sufferance: administration of treatments was performed under isoflurane anesthesia and animals were sacrificed by CO_2_ inhalation before tumour collection. Five-week old SCID/Beige mice were purchased from Harlan Laboratory. All animals were housed in sterilised laminar flow caging system and food, water and bedding were autoclaved before being put in the cages. Food and water were given *ad libitum*.

### *In vivo* efficiency of siRNA TMPRSS2-ERG IV-SQ nanoparticles

VCaP cells were *s*.*c*. inoculated [10×10^6^ cells/mouse in PBS (50 μL) mixed with Matrigel (50 μL)]. When tumours reached about 50mm^3^, mice (n = 5/group) were treated intravenously (*i*.*v*.) twice per week either with saline solution (NaCl 0.9%), non-vectorized siRNA TMPRSS2-ERG IV, siRNA Control-SQ NPs or siRNA TMPRSS2-ERG IV-SQ NPs, dispersed in 100 μL of 0.9% NaCl solution at the rate of 0.5 mg/kg for the first injection and 0.1 mg/kg for the rest of the injections (cumulative dose = 1.8 mg/kg/mouse). Mice were monitored daily for tumour growth and body weight and then sacrificed at the end of the experiment (day-40) or when tumours reached a volume of 1000 mm^3^. Tumours were immediately frozen in liquid nitrogen for Western blotting or fixed in 4% paraformaldehyde (PFA) for immunohistochemical studies (IHC).

### Protein extractions from tumours

Tumours were ground and proteins were extracted as described above. Western blot were performed to assess ERG protein level. GAPDH was used as internal control.

### Immunohistochemistry

Studies are performed as previously described by Ali et al.,[19]. Briefly, tumour tissues were embedded in paraffin, 4 μm thick sections were prepared and stained with hematoxylin-eosin-safranin (HES) and incubated with a monoclonal rabbit anti-Ki67 antibody (Lab Vision/Neomarkers, Fremont, USA, 1:200) followed by “Rabbit PowerVision Kit” (UltraVision Technologies, North Andover, USA). The signals were revealed with chemiluminescence DAB PowerVision kit (Immuno-Vision-Technologies Co., Hillsborough, USA). Sections were examined with Zeiss-Axiophot microscope (Microscopy and Imaging center, Texas, USA).

### Statistical analysis

All data are presented as mean ± standard deviation (SD). Non parametric Kruskal-Wallis analysis was used to compare multiple treatments. All pair-wise comparisons between different treatment groups were done by Tukey and Dunnet test using “R” software. *p*<0.05 was considered as a statistically significant level.

## Results

### Identification of TMPRSS2-ERG variants in VCaP cells

First, we characterised TMPRSS2-ERG transcription products in VCaP cell line that endogenously express the *TMRPSS2-ERG* fusion oncogene. Eight specifically designed primers were used to recognize the variants of TMPRSS2-ERG from I to VIII (S1 Table). As shown in Table 1, by RT-qPCR, fusion variants III and IV were found to be highly expressed in VCaP cells (Ct respectively 20 and 23), whereas variants I, II, VII and VIII were weakly expressed (Ct > 30). Fusion variants V and VI were not found to be expressed in this cell line. Since fusion variants III and IV are highly expressed in VCaP cells and correspond to the most frequent variants present in prostate cancer biopsies, they were selected for further studies involving knockdown by siRNA.

**Table 1:** Identification of fusion variants of TMPRSS2-ERG in VCaP cells by RT-qPCR analysis.

TMPRSS2-ERG fusion variants	Cycles threshold
Fusion variant I	33
Fusion variant II	32
Fusion variant III	20
Fusion variant IV	23
Fusion variant V	undetermined
Fusion variant VI	undetermined
Fusion variant VII	35
Fusion variant VIII	30
ERG WT	undetermined
TMPRSS2 WT	26
GAPDH	18

Total RNA was extracted from VCaP cells, reverse transcription was performed and cDNA was amplified by real time PCR (RT-qPCR) using primers described in S1 Table to detect TMPRSS2-ERG fusion variants.

### siRNAs TMPRSS2-ERG against variants III and IV reduce oncogene expression and affect VCaP cell viability

We designed 10 siRNAs TMPRSS2-ERG across the junction: exon 1 TMPRSS2—exon IV ERG for variant III and exon 1 TMPRSS2—exon V ERG for variant IV. According to Reynold’s rules [20], three siRNA sequences against variant III TMPRSS2-ERG and two siRNA sequences against variant IV were selected to be potentially efficient against each of the variants, as shown in the S2 Table.

The efficiency of siRNA designed against *TMPRSS2-ERG* in silencing the corresponding fusion oncogene was first tested by RT-qPCR at 24h, 48h and 72h in VCaP cells. As shown in Table 2, four siRNAs were found efficient in gene silencing [siRNA TMPRSS2-ERG III (1, 2, 3) and TMPRSS2-ERG IV (1)]. Remarkably, siRNA TMPRSS2-ERG III (1) and siRNA TMPRSS2-ERG IV (1) showed a better long-term mRNA inhibition compared to siRNA ERG (at 72h: 70% inhibition by our designed siRNA *vs* 30% inhibition by commercialized siRNA ERG). Of note, the siRNA TMPRSS2-ERG IV (1) caused the highest inhibition of TMPRSS2-ERG mRNA levels for both variants III and IV (Table 2). As expected, scramble siRNA (siRNA Control) did not show any inhibition of the fusion gene transcripts.

**Table 2:** Selection of efficient siRNA for TMPRSS2-ERG knockdown and effects on cell viability.

	Samples	TMPRSS2-ERG variant III			TMPRSS2-ERG variant IV		
		24h	48h	72h	24h	48h	72h
	**Non-treated cells**	1.0±0.11	1.0±0.20	1.0±0.09	1.0±0.18	1.0±0.19	1.0±0.09
	**siRNA Control**	1.02±0.27	1.2±0.35	1.0±0.09	1.6±0.65*	1.1±0.24	1.0±0.45
	**siRNA ERG**	0.5±0.10***	0.5±0.01***	0.6±0.02***	0.7±0.26*	0.7±0.05**	0.8±0.06***
**siRNA TMPRSS2-ERGvariant III**	**siRNA (1)**	0.6±0.14***	0.5±0.09***	0.3±0.20***	0.9±0.03	0.6±0.02***	0.5±0.01***
	**siRNA (2)**	0.5±0.16***	0.3±0.03***	0.4±0.12***	1.3±0,30	0.5±0.02***	0.8±0,25
	**siRNA (3)**	0.5±0.01*	0.6±0.11**	0.9±0.16	0.6±0.02***	0.5±0.10***	0.7±0.01**
**siRNA TMPRSS2-ERGvariant IV**	**siRNA (1)**	0.2±0.06***	0.1±0.02***	0.1±0.12***	0.5±0.13***	0.2±0.06***	0.3±0.12***
	**siRNA (2)**	0.9±0.04	1±0.06	0.9±0.04	2.3±0.78***	1.3±0.34	1.1±0.12

VCaP cells were transfected for 24h, 48h and 72h with 50 nM of siRNAs TMPRSS2-ERG III (1, 2, 3), siRNAs TMPRSS2-ERG IV (1, 2), siRNA against ERG wild type or siRNA Control. mRNA expression of TMPRSS2-ERG variants III and IV were analysed by RT-qPCR then compared to non-treated cells and results are normalised to GAPDH mRNA expression. Data are the mean of three independent experiments ± standard deviation (SD). Statistical analysis (Kruskal & Wallis followed by Tukey and Dunnet test) was performed to assess the difference between treatments compared to non-treated cells.

* = *p*<0.05,

** = *p*<0.01,

*** = *p*<0.001.

**Fig 1:**
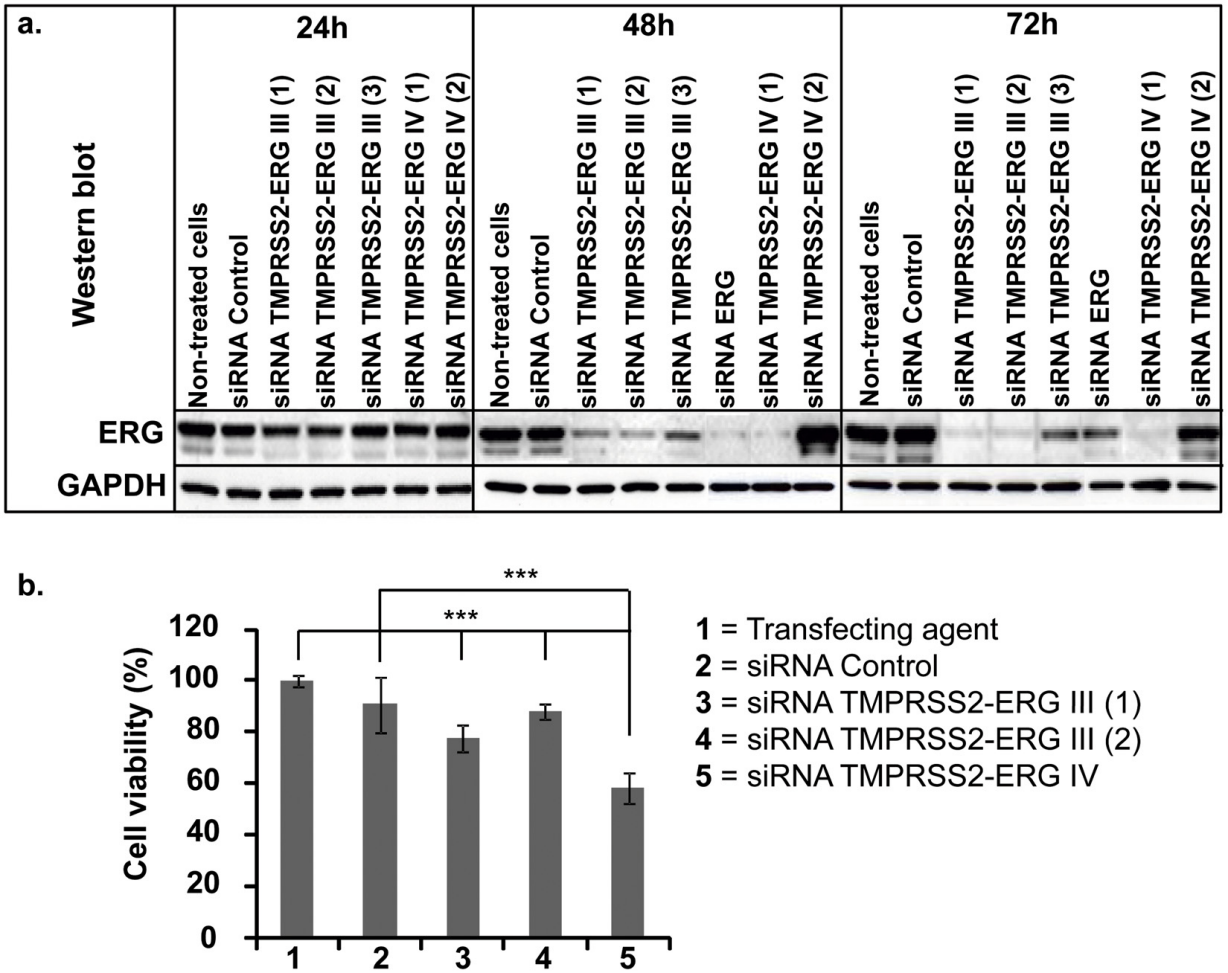
Effects of siRNA TMPRSS2-ERG on ERG protein expression and cell viability. VCaP cells were transfected for 24h, 48h and 72h with 50 nM of siRNAs TMPRSS2-ERG III (1, 2, 3), siRNAs TMPRSS2-ERG IV (1, 2), siRNA against ERG wild type or siRNA Control. **a. Western blot analysis:** the expression of ERG protein detected by Western blot showed the effects of siRNAs against TMPRSS2-ERG, ERG wild type or siRNA Control. GAPDH was used as internal control (images are representative of three independent experiments). **b. MTT cell viability assay:** VCaP cells were transfected with siRNA TMPRSS2-ERG III (1, 2), TMPRSS2-ERG IV (1) and siRNA Control at 50 nM concentration and incubated for 72h. Bars represent mean ± SD of 2 independent experiments containing 8 replicates for each condition. The number of viable cells was measured and 100% cell viability corresponds to the number of living cells incubated only with transfecting agent. *** = *p*<0.001, significant decrease compared to untreated cells or to siRNA Control (Kruskal & Wallis followed by Tukey and Dunnet test).

Then, we investigated the efficiency of siRNAs TMPRSS2-ERG on inhibition of protein expression at 24h, 48h and 72h by Western blot using ERG antibody (Fig 1a). Among the siRNAs designed against TMPRSS2-ERG variant III, both siRNAs TMPRSS2-ERG III (1) and (2) showed a better silencing efficacy than TMPRSS2-ERG III (3) and reached their maximum activity at 72h. Concerning siRNA TMPRSS2-ERG variant IV, only siRNA TMPRSS2-ERG IV (1) showed a decrease in ERG protein content. As for the previous siRNA, the highest knockdown efficiency was observed at 72h (Fig 1a).

In order to understand the biological effects of the oncogene knockdown, we assessed the viability after transfection of siRNA [siRNA TMPRSS2-ERG III (1 and 2), IV and siRNA Control] at 50 nM concentration in VCaP cells. As shown in Fig 1b, MTT assays showed a significant inhibition in growth rate for all siRNAs when compared to untreated cells (*p*<0.0001). However, only siRNAs TMPRSS2-ERG III (1) and IV (1) showed a decrease in growth rate (respectively 20% and 40%) when compared to siRNA Control (Fig 1b).

Taken together, these results demonstrate that both siRNAs TMPRSS2-ERG III (1) and TMPRSS2-ERG IV (1) are highly efficient for mRNA and protein inhibitions and to decrease the cell growth rate. Hence, these siRNAs were selected for further comprehensive investigations and onwards designated as siRNA TMPRSS2-ERG III and siRNA TMPRSS2-ERG IV, respectively.

### Low concentrations of siRNA TMPRSS2-ERG III and IV inhibit oncogene and oncoprotein expressions but did not influence cell viability

**Fig 2:**
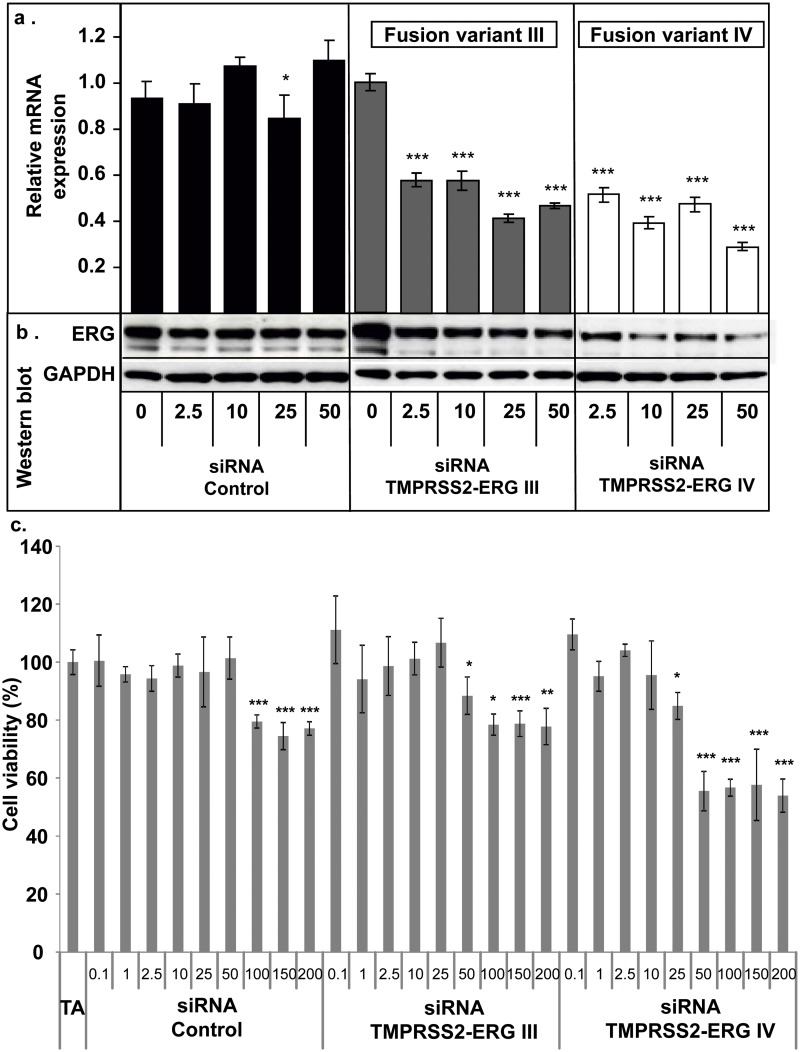
Low concentrations of siRNA TMPRSS2-ERG III and IV impair TMPRSS2-ERG oncogene and oncoprotein levels but not cell viability. VCaP cells were transfected for 72h from 2.5 nM to 50 nM with siRNA TMPRSS2-ERG III, siRNA TMPRSS2-ERG IV or siRNA Control. **a. RT-qPCR analysis:** relative TMPRSS2-ERG fusion variants III and IV mRNA levels were analysed and compared to non-treated cells. Results are normalised to GAPDH mRNA expression. Bars: mean ± SD of three independent experiments. * = *p*<0.05; *** = *p*<0.001, significant change compared to untreated cells using Kruskal & Wallis followed by Tukey and Dunnet test. **b. Western blot analysis** was used to detect the effects of siRNA TMPRSS2-ERG III, IV or siRNA Control on the expression of ERG protein. GAPDH was used as internal control (images are representative of three independent experiments). **c. MTT viability assay:** siRNA TMPRSS2-ERG III, siRNA TMPRSS2-ERG IV or siRNA Control were transfected for 72h at different concentrations (0.1 nM—200 nM). One hundred percent cell viability corresponds to the number of living cells incubated with transfecting agent only. Results are the mean ± SD of two independent experiments containing 8 replicates for each condition. * = *p*<0.05, ** = *p*<0.01, *** = *p*<0.001, significant decrease compared to untreated cells (TA) or to siRNA Control (Kruskal & Wallis followed by Tukey and Dunnet test).

In order to assess the optimal concentration to be further used for *in vivo* studies, we investigated if siRNA TMPRSS2-ERG III and IV could inhibit TMPRSS2-ERG gene and protein levels at lower concentrations than 50 nM. RT-qPCR and Western blot were performed at 48h (time corresponding to the highest down-regulation activity). Interestingly, the inhibitory effects of both siRNAs TMPRSS2-ERG were observed starting at 2.5 nM concentration (*p*<0.001, Fig 2a for RT-qPCR and 2b for Western blot). Then, cell viability was also tested after 72h transfection of siRNA TMPRSS2-ERG III, IV and siRNA Control from 0.1 to 200 nM concentrations. Of note, both siRNAs TMPRSS2-ERG III (*p*<0.05) and IV (*p*<0.001) significantly inhibited growth rate starting from 50 nM while lower concentrations did not affect cell viability and higher concentrations did not increase cell mortality (Fig 2c). Concerning the siRNA Control, a decrease in cell viability was noticed at high concentrations (100 nM, 150 nM and 200 nM), probably due to unintended toxic effects, while it does not affect cell viability at 50 nM concentration. Therefore, the 50 nM concentration seems to be the optimal dose to obtain the best specific effects in terms of gene knockdown efficiency and cell mortality (Fig 2).

### Combination of siRNA TMPRSS2-ERG III and IV did not improve knockdown efficacy and cell growth inhibition

We then assessed if the combination of both siRNAs TMPRSS2-ERG III and siRNA TMPRSS2-ERG IV improved TMPRSS2-ERG gene and protein inhibitions. VCaP cells were transfected with siRNAs TMPRSS2-ERG III and IV at a concentration of 50 nM for 72h, then mRNA and ERG protein expressions were investigated by RT-qPCR and Western blot. The combination of siRNAs TMPRSS2-ERG III and IV did not enhance the TMPRSS2-ERG gene or protein inhibitions compared to respective siRNAs alone (S1 Fig). Intriguingly, a therapeutic strategy combining both siRNAs TMPRSS2-ERG fusions III and IV did not affect cell viability compared to respective siRNAs TMPRSS2-ERG alone, as shown in the S2 Fig Therefore, siRNA TMPRSS2-ERG IV alone was found to be sufficient for ERG silencing and cell growth inhibition.

### Microarray analysis pointed out cell growth networks impairment after *TMPRSS2-ERG* oncogene silencing

Since the TMPRSS2-ERG mRNA inhibition by siRNAs TMPRSS2-ERG III and IV was found to be similar at 48h and 72h post-transfection (Table 2, compare 48h and 72h, lines 4 and 7), thus the microarray analysis was performed at 48h. First, we checked if the TMPRSS2-ERG inhibition was conserved in the three independent experiments submitted to microarray analysis and found similar results by RT-qPCR (as already observed in Table 2). For microarray analysis, a fold change of two in gene expression with a *p*-value *≤* 10^–5^ and a minimum intensity *>* 100 have been used to select changes in gene expression to restrict the study to the most affected genes. The microarray data related to the study have been submitted to the Array Express data repository at the European Bioinformatics Institute (http://www.ebi.ac.uk/arrayexpress/), under the accession number: E-MTAB-2838.

**Fig 3:**
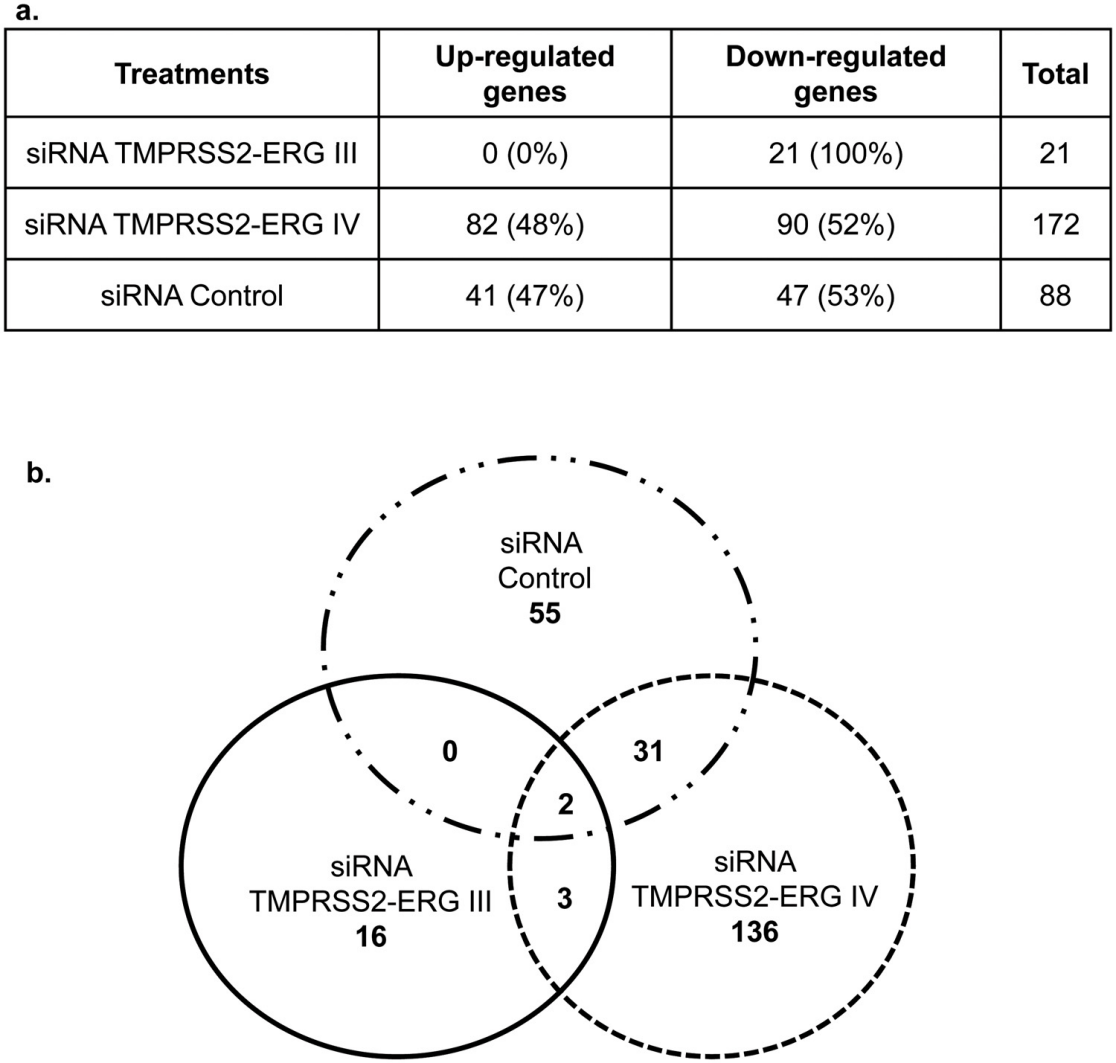
Microarray analysis of genes modulated by siRNA treatments. Agilent human whole-genome oligo microarray 8x60 k was used. Microarray images were analysed with Feature extraction software version 10.7.3.1 (Agilent). **a.** We defined up- or down-regulations as ratios greater than two-fold gene expression between VCaP cells transfected with siRNAs (TMPRSS2-ERG III, siRNA TMPRSS2-ERG IV and siRNA Control sequences) and untreated cells, acquired with a fold discovery rate ≤ 0.05 and a minimum intensity ≥ 100. **b**. Venn diagram showing the intersection between treatments using siRNA TMPRSS2-ERG III, siRNA TMPRSS2-ERG IV and siRNA Control.

Then we compared the expression profile of cells treated with siRNAs TMPRSS2-ERG III and IV to siRNA Control and detected: i) 21 of 44000 probe sets down-regulated when VCaP cells were transfected with the siRNA TMPRSS2-ERG III, ii) 172 probe sets (up or down) with the siRNA TMPRSS2-ERG IV, iii) 88 probe sets with the siRNA Control (Fig 3a). Contrary to cells treated with the siRNA TMPRSS2-ERG III (100% down-regulated), the repartition of up- or down-regulated genes is almost equally distributed when cells are transfected with siRNA TMPRSS2-ERG IV (48% up *vs* 52% down) or siRNA Control (47% up *vs* 53% down) (see Fig 3a).

We therefore crossed the data obtained for the three siRNAs (see Venn diagram, Fig 3b) and found that only three genes are co-repressed by both siRNAs against the TMPRSS2-ERG junction oncogene (*ERG*, *ADRAA2* and *SPOCK2*). By RT-qPCR, *ERG* and *ADRAA2* (a G-protein-coupled receptor) genes depicted the same down-regulation profile as in the microarray study, whereas *SPOCK2* (which regulates matrix organization) showed divergent results (Table 3, I). Two genes (*REG4* and *PHGR1*), whose biological function is unknown, were found to be regulated by siRNAs TMPRSS2-ERG (III and IV) and by siRNA Control (Fig 3b and Table 3, II). The observed down-regulation by microarray analysis was confirmed by RT-qPCR (Table 3, II). Of the 16 genes specifically down-regulated by the siRNA TMPRSS2-ERG III (Fig 3b), the first two TOP-10 genes (*ZNF32* and *SPHAR*) were assessed by RT-qPCR (Table 2, III). The down-regulation observed by microarray was confirmed when treatment by the siRNA TMPRSS2-ERG III was compared to the untreated cells, but not when compared to the siRNA Control (Table 3, III). Concerning the treatment by the siRNA TMPRSS2-ERG IV, a larger number of genes was found to be regulated with a good balance between up- and down-regulations (Fig 3a and 3b). The first TOP-10 gene (*GREM1*) found to be down-regulated in microarray analysis was detected to be up-regulated by RT-qPCR, whereas the down-regulation of the second TOP-10 gene (*PAPSS2*) was confirmed by RT-qPCR (Table 3, IV).

**Table 3:** Validation by RT-qPCR of genes found to be regulated by microarray analysis.

	*Sequence name*	*accession N°*	*Ratio untreated cells (MA and—RT-PCR)*	*Ratio siRNA Control (MA)*	*Ratio siRNA Control (Q-RT-PCR)*	*Ratio siRNA TMPRSS2-ERG III (MA)*	*Ratio siRNA TMPRSS2-ERG III (Q-RT-PCR)*	*Ratio siRNA TMPRSS2-ERG IV (MA)*	*Ratio siRNA TMPRSS2-ERG IV (Q-RT-PCR)*	*Biological function*
**(I)**	ERG	NM_004449	1	0,93	0,72 ± 0,04	0,44	0,18 ± 0,02	0,20	0,15 ± 0,04	Transcriptions factor involved in prostate carcinogenesis regulates embryonic development, cell proliferation, differentiation, apoptosis, angiogenesis, inflammation
	ADRAA2	NM_000681	1	0,97	1,85± 0,09	0,50	0,83 ± 0,13	0,36	0,75 ± 0,02	G protein-coupled receptor. Involved in aggregation, proliferation, migration, movement morphology
	SPOCK2*	NM_014767	1	0,72	1,49 ± 0,06	0,47	3,42 ± 0,21	0,35	2,13 ± 0,07	Involved in regulating matrix organization
**(II)**	REG4	NM_032044	1	0,26	0,57 ± 0,12	0,49	0,258 ± 0,01	0,15	0,21 ± 0,01	Unknown, involved inG1/S phase
	PHGR1	NM_001145643	1	0,45	0,41 ± 0,14	0,49	0,94 ± 0,2	0,28	0,68 ± 0,03	Unknown
**(III)**	ZNF32	NM_001005368	1	1,30	0,69± 0,05	0,28	0,76 ± 0,04	1,40	0,97 ± 0,07	Zinc finger protein 32 involved in regulation of transcription
	SPHAR	NM_006542	1	1,13	0,58 ± 0,03	0,32	0,56 ± 0,02	1,07	0,66 ± 0,03	Unknown, involved in DNA replication
**(IV)**	GREM1*	NM_001191323	1	0,69	0,53 ± 0,02	0,53	1,99 ± 0,03	0,24	1,447 ± 0,07	Bone morphogenic protein (BMP) antagonist family. Involved in negative regulation of bone mineralization. It is also a negative regulation of canonical Wnt receptor signaling and positively regulates angiogenesis and cell proliferation
	PAPSS2	NM_001015880	1	0,83	0,80 ± 0,02	0,52	0,27 ± 0,01	0,26	0,17 ± 0,01	Coding for Adenylyl-sulfate kinase enzyme. Involved in xenobiotic metabolism

**(I)** Genes found to be commonly regulated by siRNA TMPRSS2-ERG III and IV. **(II)** Genes found to be commonly regulated by siRNA TMPRSS2-ERG III, IV and siRNA Control. **(III)** Genes found to be specifically regulated by siRNA TMPRSS2-ERG III. **(IV)** Genes found to be specifically regulated by siRNA TMPRSS2-ERG IV.

* = genes presenting discordant results between microarray analysis and RT-qPCR. MA, microarray analysis, RT-qPCR, reverse transcription quantitative PCR.

Then, we analysed by IPA software (Ingenuity Pathway Analysis) the associated network functions for genes specifically regulated by siRNA TMPRSS2-ERG III or IV. Concerning siRNA TMPRSS2-ERG III, only one associated network involved in “cellular movement, development, growth and proliferation” was found to be relevant and accounts for six down-regulated genes (S4 Table). The siRNA TMPRSS2-ERG IV seems to be implicated in the regulation of several networks involved in cellular movement, morphology and proliferation (S4 Table). Hence, the results obtained by microarray analysis and by cell viability assays pointed out that both siRNAs TMPRSS2-ERG are involved in networks affecting cellular growth, proliferation and cell death. This prompted us to further investigate the cell death mechanism.

### Knockdown of TMPRSS2-ERG induces apoptosis

**Fig 4:**
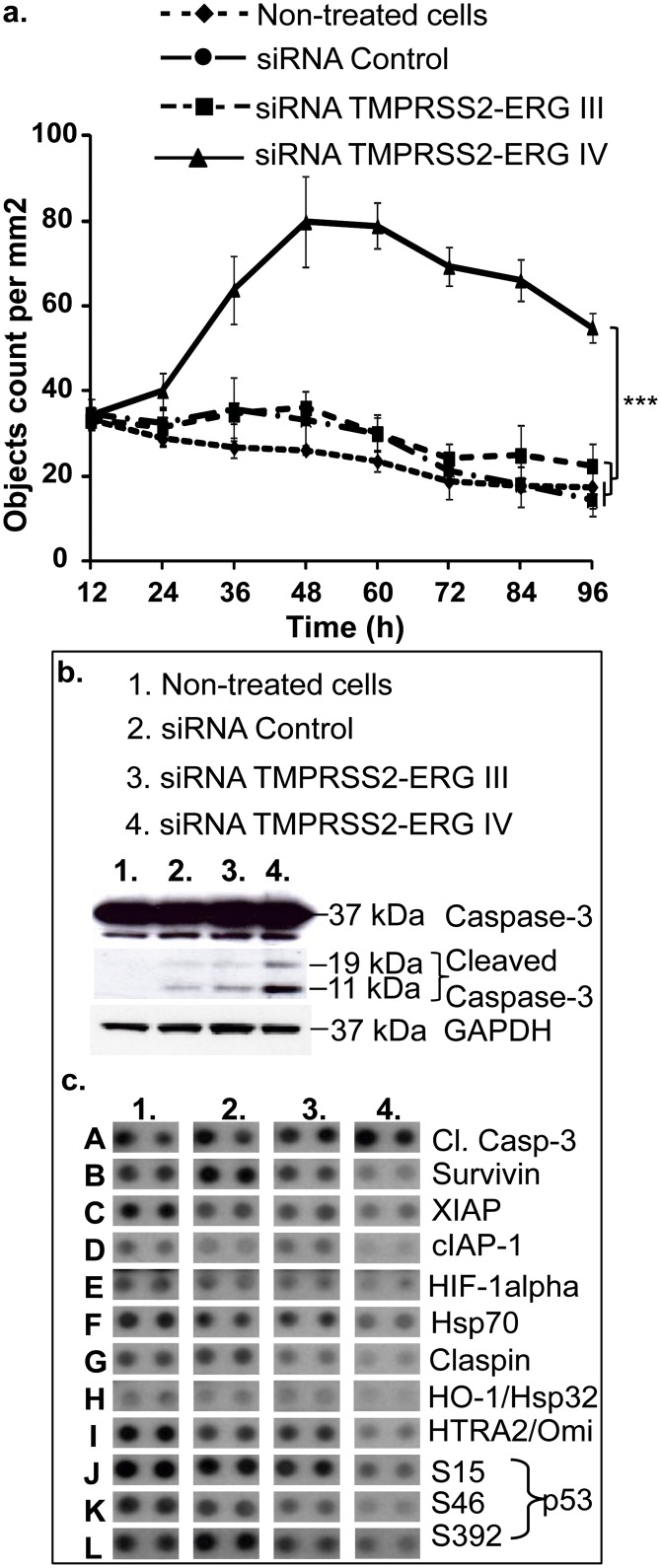
Effects of siRNA TMPRSS2-ERG III and IV on cell death pathways. **a.** VCaP cells were transfected with siRNA (TMPRSS2-ERG III, IV or siRNA Control) at 50 nM and activation of caspase-3/7 was monitored over 96 hours by IncuCytelive imaging using NucView 488 caspase-3/7 kit. The Y axis corresponds to the apoptotic fluorescent nuclei counted per mm^2^ area in each image (mean of 6 images/well). Statistical analysis (Kruskal & Wallis followed by Dunnet test) was performed to assess the difference between treatments compared to non-treated cells, *** = *p*<0.001. **b.** Western blot of cleaved caspase-3 was performed 72h after VCaP cells transfected with siRNA (TMPRSS2-ERG III, IV or siRNA Control) at 50 nM. An increase of cleaved caspase-3 is clearly observed once cells were transfected with siRNA TMPRSS2-ERG IV. **c.** Transfection of siRNAs TMPRSS2-ERG III, IV and siRNA Control was carried out in VCaP cells for 72 hours. Cells were harvested and proteins from each sample (non-treated and treated VCaP cells with siRNAs TMPRSS2-ERG III, IV or siRNA Control) were incubated overnight with the “Human Apoptosis Array” (R&D). Spots were visualized as previously described in the “Materials and Methods” section. Here we show the up- or down-regulated proteins by siRNA TMPRSS2-ERG III and IV but not by siRNA Control and compared to non-treated cells.

Annexine-V analysis showed an increase in percentage of apoptotic VCaP cells by both siRNAs TMPRSS2-ERG III and IV after 72h of transfection. However, at 96h, only the siRNA TMPRSS2-ERG IV increased its apoptotic effects while the siRNA TMPRSS2-ERG III lost it and could explain its lower efficacy in affecting VCaP cell viability (as shown in Fig 1b). The decrease of cell viability is mostly related to early and late apoptosis (Table 4, upper and lower right quadrant of each treatment in S3 Fig) and not due to necrosis (Table 4, upper left quadrant of each treatment in S3 Fig). To decrypt the partner proteins involved in cell death due to apoptosis, we first analysed cleavages of caspases-3 and -7 by IncuCyteimaging system using NucView488 kit (Fig 4a). When cells were transfected with siRNA TMPRSS2-ERG IV, an increase of caspases-3 and -7 cleavages was observed, whereas the siRNA TMPRSS2-ERG III did not show any statistical difference compared to either siRNA Control or to untreated cells (Fig 4a). These results were confirmed by Western blot analysis wherein only siRNA TMPRSS2-ERG IV showed an increase in caspase-3 cleavage at 72h (Fig 4b). Then a wider screening of apoptosis-related proteins was performed at 72h using the “Proteome Profiler Array”. This allowed us to confirm the results obtained by IncuCyte and Western blot for caspase cleavage (Fig 4c, line A), and to detect modifications of protein expression in 11 of the 35 spotted proteins due to TMPRSS2-ERG knockdown by both siRNA (Fig 4c). Contrary to cleaved caspase-3, which was found to be increased (Fig 4c, line A), all the other modified proteins were decreased by both siRNAs TMPRSS2-ERG (Fig 4c, lines B to L). Interestingly, proteins known to counteract caspase-3 cleavage (survivin, XIAP, cIAP-1, HIF-1α and Hsp70) were found to be negatively regulated by both siRNA TMPRSS2-ERG but with a more pronounced inhibition by the siRNA designed against the variant IV (Fig 4c, lines B to F). Moreover, claspin and HO-1/Hsp32, proteins involved in oxidative stress, were also inhibited mainly by the siRNA TMPRSS2-ERG IV (Fig 4c, lines G and H). Concerning HTRA2/Omi, a serine protease like TMPRSS2, a strong inhibition was observed mostly by the siRNA TMPRSS2-ERG IV (Fig 4c, line I). Unexpectedly, p53 phosphorylated serines were found to be inhibited by both siRNAs and particularly by the siRNA TMPRSS2-ERG IV (Fig 4c, lines J to L).

**Table 4:** Effects of siRNA TMPRSS2-ERG III and IV on cell death pathways by flow cytometry.

Incubation time	Treatments	live cells	early apoptosis	late apoptosis	dead cells
		Annexine V^-^/PI^-^	Annexine V^+^/PI^-^	Annexine V^+^/PI^+^	Annexine V^-^/PI^+^
**72 h**	Non-treated cells	70%	10%	11%	9%
	siRNA Control	70%	9%	9%	12%
	siRNA TMPRSS2-ERG III	59%	22%	17%	2%
	siRNA TMPRSS2-ERG IV	53%	20%	19%	8%
**96 h**	Non-treated cells	80%	5%	6%	9%
	siRNA Control	84%	2%	2%	12%
	siRNA TMPRSS2-ERG III	75%	9%	7%	9%
	siRNA TMPRSS2-ERG IV	15%	59%	21%	5%

VCaP cells were transfected with siRNA (TMPRSS2-ERG III, IV) or siRNA Control at 50 nM concentration for 72h and 96h. Cells were incubated with Annexin-V-Fluos and propidium iodide and analysed by flow cytometry. Results represent the percentage of cells differently stained and corresponding to: living cells (AV^-^/PI^-^), early apoptotic cells (AV^+^/PI^-^), late apoptotic cells (AV^+^/PI^+^) and dead cells (AV/PI^+^).

Taking together these results on cell viability and apoptosis, siRNA TMPRSS2-ERG IV seems to be the best candidate with the highest therapeutic potential. It was thus selected for linkage with squalene for vectorisation by “squalenoylation” technology, allowing further *in vitro* and *in vivo* investigations.

### NPs siRNA TMPRSS2-ERG IV-SQ are only efficient in preclinical studies

In order to protect the siRNAs from degradation, squalene (SQ) was coupled covalently to siRNA TMPRSS2-ERG IV and to siRNA Control. In water, both bio-conjugates self-assembled spontaneously into nanoparticles (NPs) of about 190 nm of diameter, with a poly-dispersity index of 0.18 and a Zeta potential of ~ -27 mV.

As shown in S4 Fig, upper panel, NPs siRNA-SQ were unable to enter within the cells without a transfecting agent. However, when NPs siRNA TMPRSS2-ERG-SQ were transfected with the cationic lipid (Lipofectamine RNAiMAX), a decrease of about 70% of TMPRSS2-ERG mRNA expression was observed. A high reduction in protein content was paralleled with decreased TMPRSS2-ERG mRNA expression levels (S4 Fig, lower panel). This demonstrates that the siRNA TMPRSS2-ERG IV is still active after bio-conjugation with squalene, due to modification only in the passenger sense strand and the use of an ester hydrolysable bond between the squalene moiety and the siRNA part.

**Fig 5:**
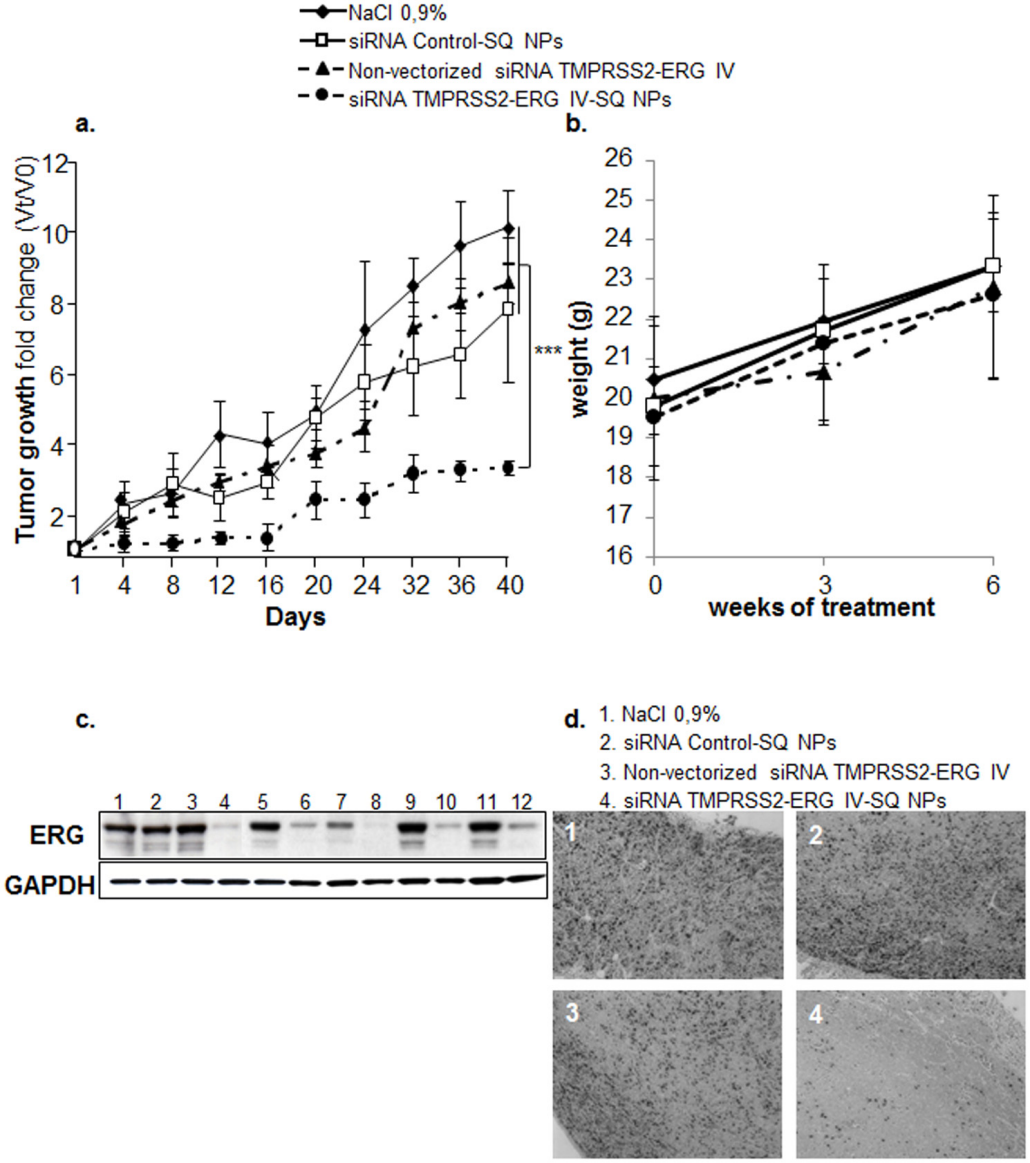
Vectorized siRNA TMPRSS2-ERG IV-SQ impaired tumour growth and restored differentiation *in vivo*. **a.** SCID mice (n = 5/group) bearing VCaP tumour xenografts were injected intravenously either with NaCl 0.9%, non-vectorized siRNA TMPRSS2-ERG IV, NPs siRNA control-SQ or NPs siRNA TMPRSS2-ERG IV-SQ twice per week during 6 weeks (1.8 mg/kg/mouse cumulative dose). The tumour growth was followed during the course of the experiment (until day 40). Using the Kruskal & Wallis followed by Tukey and/or Dunnet test, a statistical decrease (*** = *p*<0.001) of tumour growth was observed when mice were treated with siRNA TMPRSS2-ERG IV-SQ NPs. **b.** The weight of mice was monitored regularly from the beginning to the end of the treatment. No statistical difference in weight evolution was observed between the first and the last treatment and also among the different treatments. **c**. Mice were sacrificed and tumours were collected at the end of the experiment and ERG protein expression was analysed by Western blot. GAPDH was monitored as loading control. Lines 1, 5, 7, 9 and 11 correspond to 5 independent tumours treated with NaCl 0.9%; line 2 corresponds to tumour treated with siRNA Control-SQ NPs, line 3 to unvectorized siRNA TMPRSS2-ERG and lines 4, 6, 8, 10 and 12 correspond to 5 independent tumours treated with siRNA TMPRSS2-ERG-SQ NPs. **d.** Immunohistochemical analysis revealed a decreased Ki67 positive nuclei only in the tumours treated with NPs siRNA TMPRSS2-ERG IV-SQ. Photograph magnification is 20X.

As shown in Fig 5a, when the NPs siRNA TMPRSS2-ERG-SQ were injected by *i*.*v*. in SCID mice, tumour growth was strikingly inhibited (70%, *p*<0.001) compared to mice treated with NPs siRNA Control-SQ, non-vectorized siRNA TMPRSS2-ERG IV or saline solution. Neither mouse viability nor weight loss was observed in any group regardless of the treatments administered (Fig 5b). Tumours collected at the end of experiments were analysed for the expression of ERG oncoprotein by Western blot and the results revealed a high reduction of ERG protein levels (Fig 5c). Moreover, Ki67 proliferation marker was also decreased in tumours treated with NPs siRNA TMPRSS2-ERG IV-SQ (Fig 5d), indicating a reduction of mitotic index by vectorized siRNA TMPRSS2-ERG treatment.

## Discussion

The discovery of the chromosomal rearrangement leading to *TMPRSS2-ERG* fusion oncogene, present in more than 50% cases of prostate cancers, has opened important diagnostic and therapeutic perspectives for the treatment of one of the most frequent epithelial tumours in men. The aim of this study was to conceive a pharmacological approach targeting *TMPRSS2-ERG* fusion oncogene by therapeutic siRNA.

To achieve this goal, we first selected the most representative cell line for this neoplastic pathology. According to the literature, only two cell-lines, VCaP and NCI-H660, have been shown to express *TMPRSS2-ERG* fusion oncogene, but only the VCaP cells were found to be tumorigenic in immune-repressed mice [24]. Furthermore, in our experiments, the VCaP cell line was found to be representative of the prostate cancer biopsies, since it expressed the most frequent variants of *TMPRSS2-ERG* fusion oncogene found in patients, namely variants III and IV [16]. Therefore, to introduce a personalised treatment for patients with prostate cancer, we focused our targeted therapy by silencing TMPRSS2-ERG variants III and IV. Of the five designed siRNA (three against variant III and two against variant IV), three were found to inhibit both TMPRSS2-ERG mRNA and ERG protein levels to more than 50% at 50 nM concentration with an overtime lasting effect. In addition, cell viability was also impaired with a more pronounced effect when VCaP cells were transfected with siRNA TMPRSS2-ERG III (1) and IV (1) (labelled, from now on, siRNA TMPRSS2-ERG III and IV). However, siRNA concentrations lower or higher than 50 nM and combination of both siRNAs TMPRSS2-ERG III and IV did not improve knockdown efficacy or diminish the VCaP cell viability. We thus speculated that lower siRNA concentrations would not allow a long-lasting efficacy required to obtain a biological effect, while higher concentrations (above 50 nM) would favour unspecific effects such as an unintended decrease in cell viability as observed for the siRNA Control.

When both siRNAs TMPRSS2-ERG (III and IV) were combined, the absence of synergistic activity (gene knockdown and cell viability) might be due to the saturation of the RISC machinery whose enzymatic activity could not be further improved. Although this finding might seem to be a negative result, indeed from the pharmaceutical point of view, the development of only one therapeutic siRNA with the same efficiency than several combined, is certainly simpler and safer, thus, easier to progress towards clinical investigations.

To identify the genes affected by the *TMPRSS2-ERG* junction oncogene inhibition, a comprehensive transcriptome analysis (microarray) was performed on mRNA isolated from VCaP cells treated either with siRNA TMPRSS2-ERG III, IV or siRNA Control. An equilibrium between up- and down-regulated genes was observed for siRNA TMPRSS2-ERG IV and siRNA Control, but the biological function of the genes is quite different. In fact, the siRNA Control did not regulate any specific cluster of genes whereas, genes regulated by TMPRSS2-ERG type IV essentially belong to known networks of cellular movement, development, growth, death and proliferation. Interestingly, these genes were found to be different from those regulated by the androgen receptor [25], the main target for prostate cancer treatment. This suggests that proliferation may be stimulated by AR independent mechanisms in castration resistant prostate cancer through TMPRSS2-ERG activation.

Surprisingly, the siRNA TMPRSS2-ERG III showed only down-regulations, suggesting an off-target effect; however, the biological function of these genes partially discarded this hypothesis since most of them are involved in cellular movement, development, growth and proliferation networks. RT-qPCR analysis of genes regulated by each treatment alone or co-regulated by the different treatments confirmed the microarray profile of seven out of nine tested genes. Indeed, only three genes were found to be co-regulated by both therapeutic siRNAs (TMPRSS2-ERG III and IV). This could be due to the fact that each variant might independently regulate different genes. Similarly, Wang *et al*. described different biological effects after transfection of variant III or variant VI in the same cell line suggesting separate roles of each variants within the cell [13]. Thus, inhibiting one transcript does not necessarily affects the genes regulated by the other one. Therefore, we focused on the two validated genes (*ERG*, *ADRAA2*) co-regulated by therapeutic siRNA. The down-regulation of ERG by both siRNAs TMPRSS2-ERG confirms the validity of microarray results. Concerning *ADRAA2*, this gene is described to be involved in aggregation, proliferation and migration. Its decrease could have a biological significance and may represents a direct effect of the *TMPRSS2-ERG* junction oncogene silencing by siRNA, but further studies are required to test this hypothesis. Taking together, the microarray results showed that the junction oncogene *TMPRSS2-ERG* confers a survival phenotype to cells and its inhibition could be responsible for the observed decrease in cell viability. Additionally, this was confirmed by cell death studies where early and intermediate apoptosis was detected and found to be more pronounced in siRNA TMPRSS2-ERG IV treated cells. A complementary approach was used to identify proteins that are predominantly affected during apoptosis by proteomic array. The apoptosis proteomic profile showed increasing levels of cleaved caspase-3 due to a reduction of its partner proteins (survivin, XIAP, cIAP, HIF-1α, Hsp70), suggesting a key role of caspase-3 in apoptosis occurrence. Moreover, TMPRSS2-ERG knockdown by therapeutic siRNA inhibits claspin and HO-1/Hsp32 proteins, sensitizing cells to oxidative stress and therefore to apoptosis, probably through accumulation of Reactive Oxygen Species (ROS).

Surprisingly, a reduction of HTRA2/Omi and phosphorylation of p53 (serines S15, S46 and S392) levels were observed when cells were treated with siRNA TMPRSS2-ERG. HTRA2/Omi was described to be a pro-apoptotic protein but its expression promotes prostate cancer dedifferentiation [26]. Serine phosphorylation of p53 was observed during apoptosis [27], but the phosphorylation of p53 (S392) has an antiapopototic effect and promotes cell survival [28, 29]. This would represent a double-edged sword for prostate cancer survival and progression, as the inhibition observed could favor resistance to apoptosis by siRNA TMPRSS2-ERG treatment or decrease dedifferentiation and therefore enhance cancer cell death. Further studies are needed to investigate the physiological significance of the observed HTRA2/Omi and p53 phosphorylation inhibition by therapeutic siRNA. Moreover, these studies point out the importance to combine microarray and proteomic approaches to identify proteins found to be unaffected in their mRNA levels by microarray analysis.

Taken together, our results suggest that siRNA TMPRSS2-ERG IV is the best candidate for therapeutic purposes, and therefore we used squalene, a natural and nonionic lipid, to deliver siRNA (TMPRSS2-ERG IV and Control) [30]. This vectorisation method was employed because this triterpene is known for its biocompatibility and inertness so that it is already extensively used as an excipient in numerous pharmaceutical formulations [31]. Chemical conjugation of siRNA TMPRSS2-ERG IV to squalene improves siRNA hydrophobicity and stability. As previously described, the siRNA can self-aggregate as nanoparticles in aqueous solution when covalently bound to squalene and found to be inefficient *in vitro* if they are not transfected with a cationic compound [19, 32, 33]. Interestingly, when mice bearing prostate VCaP xenografted cells were treated with NPs siRNA TMPRSS2-ERG IV-SQ, the tumour growth was strikingly inhibited as early as the first week of treatment. Within the tumours, a strong inhibition of ERG oncoprotein was detected. Moreover, the treatment was able to partially restore differentiation (decrease of Ki67 marker) without any signs of toxicity. It should be noticed that the injected dose of our designed siRNA TMPRSS2-ERG IV-SQ NPs was very low compared to several other siRNA nano-medicines [34, 35]. These data demonstrate once again that the antineoplastic disparity observed between *in vitro* and *in vivo* studies may be due to the difference in enzymatic contents leading to changes in physicochemical properties of the nanoparticles and influencing the kinetic release of the siRNA from the squalenoylated nanoparticles.

Several studies already demonstrated the importance of knocking down ERG over-expression by siRNA in prostate cancer [13, 36–38]; one of them has suggested the targeting of TMPRSS2-ERG fusion with siRNA delivered *via* liposomal nanovectors. In our case, contrary to many nanosystems, wherein a significant quantity of lipids or polymers is required to encapsulate the active principle, the use of a covalent but hydrolysable bond between the molecule and vehicle authorizes the use of much lower amounts of vehicle, without decreasing drug concentrations and compromising the stability of the nanosystem.

In conclusion, we used an experimental approach that mimics the prostate tumours in their physiological environment and points out a concrete clinical application for prostate cancer therapy based on TMPRSS2-ERG knockdown. Moreover, knockdown of TMPRSS2-ERG by siRNA inhibits growth and proliferation independently from AR-signaling pathway. Therefore, siRNA TMPRSS2-ERG-squalene nanoparticles may be a promising alternative therapy for patients with castration resistant prostate cancer.

## Supporting Information

S1 Table Sequences of primers designed for TMPRSS2-ERG variants (I to VIII) and for ERG and TMPRSS2 wild types. (PDF)

S2 Table Sequences of siRNA TMPRSS2-ERG designed across fusion variant III and fusion variant IV. For the siRNA Control: bases underlined correspond to the five mismatches introduced in the sequence of the siRNA TMPRSS2-ERG IV (1).
(PDF)

S3 Table Sequences of primers designed for genes found to be regulated in microarray analysis and validated by RT-qPCR analysis. (PDF)

S4 Table Genes found to be specifically regulated by siRNA TMPRSS2-ERG III or siRNA TMPRSS2-ERG IV and involved in networks affecting cellular movement, survival, and morphology (1 of 2 pages). (PDF)

S1 Fig Inhibitory effects of the combination of siRNAs TMPRSS2-ERG III and IV in oncogene and oncoprotein expressions. VCaP cells were transfected for 72h with siRNAs alone (TMPRSS2-ERG III, IV or Control) or in combination (TMPRSS2-ERG III and IV) at 50 nM concentration. For RT-qPCR analysis, cells were harvested, mRNA extracted and RT-qPCR performed. Relative TMPRSS2-ERG fusion variants III and IV mRNA levels were analysed then compared to non-treated cells and results are normalised to GAPDH mRNA expression. Bars represent the mean ± SD of three independent experiments. Using Kruskal & Wallis test followed by Tukey tests, a statistical difference was observed between treatments compared to non-treated cells: *** = *p*<0.001. For Western blot analysis, ERG protein level in VCaP cells were analysed after 72h of treatment. GAPDH was used as loading control. The figure shows one representative of three independent experiments.
(PDF)

S2 Fig Inhibitory effects of the combination of siRNAs TMPRSS2-ERG III and IV on cell viability. VCaP cells were transfected for 72h with siRNAs alone (TMPRSS2-ERG III, IV or Control) or in combination (TMPRSS2-ERG III and IV) at 50 nM concentration and the MTT viability assay was performed. The number of viable cells was measured and compared to non-treated cells incubated with transfecting agent only (100% cell viability). Results are the mean ± SD of two independent experiments containing 8 replicates for each condition. Statistical analysis (Kruskal & Wallis followed by Tukey test) was performed to assess the difference between treatments compared to non-treated cells. ** = *p*<0.01, *** = *p*<0.001.
(PDF)

S3 Fig Effects of siRNA TMPRSS2-ERG III and IV on cell death pathways by flow cytometry. (PDF)

S4 Fig Upper panel: uptake of Nanoparticles in VCaP cell line. The intracellular localisation of FAM-labelled nanoparticles was assessed by using fluorescence microscope. VCaP cells were treated with 50 nM of NPs FAM-labelled siRNA TMPRSS2-ERG IV-SQ in the presence or absence of Lipofectamine RNAiMAX and incubated at 37°C for 24 h. Cell nuclei were stained with DAPI (blue) and FAM-labelled siRNA TMPRSS2-ERG tumour (green) were observed with fluorescence microscope at 20X. **Lower panel: inhibition of TMPRSS2-ERG oncogene and oncoprotein by NPs**. VCaP cells were transfected for 48h with FAM-labelled siRNA TMPRSS2-ERG IV-SQ NPs in the presence or absence of transfecting agent Lipofectamine RNAiMAX (TA). Cells were then harvested and relative mRNA and ERG protein levels were analysed by RT-qPCR and Western blot respectively. Treatments correspond to: 1. Non-treated cells, 2. siRNA TMPRSS2-ERG IV-SQ NPs in the absence of transfecting agent, 3. siRNA TMPRSS2-ERG IV-SQ NPs in the presence of transfecting agent, 4. siRNA TMPRSS2-ERG IV in the presence of transfecting agent.
(PDF)
