# Supplementary material for: Antineoplastic Effects of siRNA against TMPRSS2-ERG Junction Oncogene in Prostate Cancer
Source: PLoS One. 2015 May 1;10(5):e0125277. doi: 10.1371/journal.pone.0125277 (PMC4416711; doi:10.1371/journal.pone.0125277)
Supplement: S1 Table — (PDF) [file pone.0125277.s001.pdf]

S1 Table. Sequences of primers designed for TMPRSS2-ERG variants (I to VIII) and for ERG and TMPRSS2 wild types.

Primers	Forward = F<br>Reverse = R	Sequences (5'-3')
TMPRSS2-ERG Variant I	F	CGGCAGGTTATTCCAGGAT
	R	CACTCACAACTGATAAGGCTTC
TMPRSS2-ERG Variant II	F	GCAGCCGTCAGGTTCTGAA
	R	TTGGAAGTCTGTCCATAGTCGC
TMPRSS2-ERG Variant III	F	CGGCAGGAAGCCTTATCAGT
	R	AGTTCATCCCAACGGTGTCTG
TMPRSS2-ERG Variant IV	F	GGCAGGAACTCTCCTGAT
	R	CGTGGCACGATAACTCTG
TMPRSS2-ERG Variant V	F	CTTTGAACTCAGTTATTCCA
	R	ATCTCTGTCTTAGCCAGGT
TMPRSS2-ERG Variant VI	F	GATGGCTTTGAACTCAGAAG
	R	CTGGCTAGGGTTACATTCC
TMPRSS2-ERG Variant VII	F	CTTTGAACTCAGAACTCTCCT
	R	GGTCTGTACTCCATAGCGTAG
TMPRSS2-ERG Variant VIII	F	GCACCTCAAGAAGCCTTATC
	R	GTTGAGACAGCCAATCCTG
ERG wild type	F	TTTCACTTGGTCGGAATGGG
	R	TGCTTTTGGTCAACACGGC
TMPRSS2 wild type	F	AAGGGAAGACCTCAGAAGTG
	R	CCAGTCCGTGAATACCATC
GADPH	F	CCACTCCTCCACCTTTGAC
	R	ACCCTGTTGCTGTAGCCA
