# Supplementary material for: Antineoplastic Effects of siRNA against TMPRSS2-ERG Junction Oncogene in Prostate Cancer
Source: PLoS One. 2015 May 1;10(5):e0125277. doi: 10.1371/journal.pone.0125277 (PMC4416711; doi:10.1371/journal.pone.0125277)
Supplement: S2 Table — For the siRNA Control: bases underlined correspond to the five mismatches introduced in the sequence of the siRNA TMPRSS2-ERG IV (1). (PDF) [file pone.0125277.s002.pdf]

S2 Table.

Sequences of siRNA TMPRSS2-ERG designed across fusion variant III and fusion variant IV.

**siRNA**	**Strand**	**Sequences (5'-3')**
siRNA TMPRSS2-ERG III (1)	Sense	GCA GGA AGC CUU AUC AGU UGU
	Anti-sense	AAC UGA UAA GGC UUC CUG CCG
siRNA TMPRSS2-ERG III (2)	Sense	GCG GCA GGA AGC CUU AUC AGU
	Anti-sense	UGA UAA GGC UUC CUG CCG CGC
siRNA TMPRSS2-ERG III (3)	Sense	AGC GCG GCA GGA AGC CUU AUC
	Anti-sense	UAA GGC UUC CUG CCG CGC UCC
siRNA TMPRSS2-ERG IV (1)	Sense	GCA GGA ACU CUC CUG AUG AAU
	Anti-sense	UCA UCA GGA GAG UUC CUG CCG
siRNA TMPRSS2-ERG IV (2)	Sense	GGA GCG CGG CAG GAA CUC UCC
	Anti-sense	AGA GUU CCU GCC GCG CUC CAG
siRNA Control	Sense	GCA GGA CCU AUC CCG AUA AUU
	Anti-sense	UUA UCG GGA UAG GUC CUG CUU

For the siRNA Control: bases underlined correspond to the five mismatches introduced in the sequence of the siRNA TMPRSS2-ERG IV (1).
