# Supplementary material for: Antineoplastic Effects of siRNA against TMPRSS2-ERG Junction Oncogene in Prostate Cancer
Source: PLoS One. 2015 May 1;10(5):e0125277. doi: 10.1371/journal.pone.0125277 (PMC4416711; doi:10.1371/journal.pone.0125277)
Supplement: S3 Table — (PDF) [file pone.0125277.s003.pdf]

S3 Table. Sequences of primers designed for genes found to be regulated in microarray analysis and validated by RT-qPCR analysis.

Primers	Forward = F<br>Reverse = R	Sequences (5'→3')
ADRAA2	F	CGTGGTCATCGGAGTGTTC
	R	GCGGAAATCGTGGTTGAAG
SPOCK2	F	TAACCCAAGGCGGAGAATG
	R	CACGAGACCCAGAGAAAAGG
REG4	F	GTCGAGGAACTGGTCTGATG
	R	ATATCGGCTGGCTTCTCTG
PHFR1	F	CCACATCACTGAGGAAGTAG
	R	GGAGGCTGTTTATTAGGAAG
ZNF32	F	CAAGGAGTGTGGGAAAAG
	R	CTGTGTGGACTCTGATGTG
SPHAR	F	TCTTCATCCAGGTCAGTTC
	R	TTCCAGAAAAAGCACAGTAC
GREM1	F	AGAGAAGACGACGAGAGTAAG
	R	AACCAGTAGCAGATGAACAG
PAPSS2	F	CAGCAGAAATCCACCAATG
	R	TCCAGGGACAAACTTATCG
