# Supplementary material for: Antineoplastic Effects of siRNA against TMPRSS2-ERG Junction Oncogene in Prostate Cancer
Source: PLoS One. 2015 May 1;10(5):e0125277. doi: 10.1371/journal.pone.0125277 (PMC4416711; doi:10.1371/journal.pone.0125277)
Supplement: S4 Table — (PDF) [file pone.0125277.s004.pdf]

S4 Table. Genes found to be specifically regulated by siRNA TMPRSS2-ERG III or siRNA TMPRSS2-ERG IV and involved in networks affecting cellular movement, survival and morphology (1 of 2 pages)

siRNA	***Networks***	***Symbol***	***Gene name***	***Biological Function***	***Fold Change***
**siRNA TMPRSS2-ERG III**	*Cellular movement, Cellular Development, Cellular Growth and Proliferation*	NPR3	natriuretic peptide receptor C/guanylate cyclase C	Involved in proliferation, abnormal morphology	-2,565
		ERG	v-ets avian erythroblastosis virus E26 oncogene homolog	Involved in proliferation, apoptosis	-2,278
		EBAG9	estrogen receptor binding site associated, antigen, 9	Involved in abnormal morphology, apoptosis, transmembrane potential	-2,270
		PGAM1	phosphoglycerate mutase 1	Involved in metabolic processes	-2,099
		PSMB5	Proteasome subunit, beta type, 5	Involved in senescence,survival in cancer	-2,088
		P2RY1	purinergic receptor P2Y, G-protein coupled, 1	Involved in aggregation, cell spreading	-2,041
**siRNA TMPRSS2-ERG IV**	*Cell Morphology, Cell-To-Cell Signaling and Interaction, Cell Signaling*	ERG	v-ets avian erythroblastosis virus E26 oncogene homolog	Transcription factor involved in prostate cancerogenesis: regulates embryonic development, cell proliferation, differentiation, angiogenesis, inflammation, and apoptosis.	-5,101
		MGLL	monoglyceride lipase	Enzyme involved in colony formation, migration, viral entry	-2,815
		LPXN	leupaxin	Involved in cell spreading	-2,291
		PDHB	pyruvate dehydrogenase beta	Mutations in this gene are associated with pyruvate dehydrogenase E1-beta deficiency	-2,089
		OXSR1	oxidative stress responsive 1	Involved in regulation of actin cytoskeleton	-2,060
		TIAM1	T-cell lymphoma invasion and metastasis 1	Involved in migration, invasion, adhesion, outgrowth, proliferation, apoptosis, growth, cell spreading, cell rounding	-2,025
		GAK	cyclin G associated kinase	Involved in growth, replication	2,060
		RPS4X	ribosomal protein S4, X-linked	Involved in RNA metabolic process	2,074
		ZNF692	zinc finger protein 692	Involved in regulation of transcription	2,147
		RPS27	ribosomal protein S27	Involved in RNA metabolic process	2,293
		SNHG17	small nucleolar RNA host gene 17 (non-protein coding)	Unknown	2,313
		LAT	linker for activation of T cells	Involved in immune response; immune system process, Fc-epsilon receptor signaling pathway	2,437
	Tissue Morphology, Hematological System Development and Function, Cellular Function and Maintenance	ANXA1	annexin A1	Involved in adhesion, proliferation, apoptosis, infiltration, migration, cell cycle progression	-3,176
		CALCA	calcitonin-related polypeptide alpha	Involved in proliferation, migration, apoptosis, ossification, differentiation, homeostasis	-2,662
		ADRA2A	adrenoceptor alpha 2A	Involved in aggregation, proliferation, migration, organization, movement, shape change, morphology	-2,651
		HLA-DMB	major histocompatibility complex, class II, DM beta	Involved in antigen processing and presentation	-2,620
		SLC31A2	solute carrier family 31, member 2	Accumulation of this protein has been observed in schitzofrenia	-2,183
		PIDD	p53-induced death domain protein	Involved in apoptosis, sumoylation, ubiquitination, cell death, cytostasis	2,011
		PYGM	phosphorylase, glycogen, muscle	Involved in homeostasis and proliferation in McArdle's syndrome	2,042
		AGER	advanced glycosylation end product-specific receptor	Involved in proliferation, apoptosis, migration, chemoattraction, morphogenesis	2,117
		REC8	REC8 meiotic recombination protein	Involved in meiosis, maturation, cohesion	2,287

		RNU2-2P	RNA, U2 small nuclear 2, pseudogene	Unknown	2,848
		TNFRSF25	tumor necrosis factor receptor superfamily, member 25	Involved in apoptosis, cell death, growth, polarization	2,065
**siRNA TMPRSS2-ERG IV**	*Cellular Growth and Proliferation, Gene Expression, Cell Cycle*	CRABP2	cellular retinoic acid binding protein 2	Involved in cytostasis, growth, apoptosis, proliferation	-2,660
		UCHL3	ubiquitin carboxyl-terminal esterase L3	Involved in abnormal morphology, differentiation, proliferation	-2,446
		SRPR	signal recognition particle receptor	Involved in cellular protein metabolic process	-2,219
		CUX1	cut-like homeobox 1	Involved in differentiation, cell survival, G1 phase, apoptosis, mitosis	-2,089
		CCNH	cyclin H	Involved in proliferation, cell cycle progression, migration, invasiveness	-2,082
		VWF	von Willebrand factor	Involved in aggregation, adhesion, cell spreading	-2,041
		ACKR3	atypical chemokine receptor 3	Involved in survival, migration, chemotaxis, proliferation, growth, sphere formation	-2,020
		HELZ	helicase with zinc finger	Helicase activity	-2,010
		PPT2	palmitoyl-protein thioesterase 2	Its lack leads to abnormal morphology, ataxia	2,119
		CSAD	cysteine sulfinic acid decarboxylase	metabolic processes	2,169
		NEIL1	nei endonuclease VIII-like 1	Involved in survival, proliferation	2,276
	Cellular Movement, Cancer, Cellular Development	REG4	regenerating islet-derived family, member 4	Involved in G1/S phase transition	-6,616
		PAPSS2	3'-phosphoadenosine 5'-phosphosulfate synthase 2	Involved in blood coagulation, bone development, metabolic processes	-3,889
		MEST	mesoderm specific transcript	Involved in enlargement, growth	-2,591
		GLS	glutaminase	Involved in synaptic transmission	-2,522
		HS2ST1	heparan sulfate 2-O-sulfotransferase 1	Involved in metabolic processes	-2,299
		ARHGDIB	Rho GDP dissociation inhibitor (GDI) beta	Involved in proliferation, movement, organization, adhesion	-2,257
		LPP	LIM domain containing preferred translocation partner in lipoma	Involved in migration, cell spreading, transformation, differentiation	-2,097
		MT-ND4	NADH dehydrogenase, subunit 4	NADH dehydrogenase activity	2,049
		CBFA2T2	core-binding factor, runt domain, alpha subunit 2; translocated to, 2	Involved in epithelial cell differentiation	2,091
		MT-ND5	NADH dehydrogenase, subunit 5	Involved in response to hypoxia	2,176
