# Supplementary material for: Antineoplastic Effects of siRNA against TMPRSS2-ERG Junction Oncogene in Prostate Cancer
Source: PLoS One. 2015 May 1;10(5):e0125277. doi: 10.1371/journal.pone.0125277 (PMC4416711; doi:10.1371/journal.pone.0125277)
Supplement: S1 Fig — VCaP cells were transfected for 72h with siRNAs alone (TMPRSS2-ERG III, IV or Control) or in combination (TMPRSS2-ERG III and IV) at 50 nM concentration. For RT-qPCR analysis, cells were harvested, mRNA extracted and RT-qPCR performed. Relative TMPRSS2-ERG fusion variants III and IV mRNA levels were analysed then compared to non-treated cells and results are normalised to GAPDH mRNA expression. Bars represent the mean ± SD of three independent experiments. Using Kruskal & Wallis test followed by Tukey tests, a statistical difference was observed between treatments compared to non-treated cells: *** = p<0.001. For Western blot analysis, ERG protein level in VCaP cells were analysed after 72h of treatment. GAPDH was used as loading control. The figure shows one representative of three independent experiments. (PDF) [file pone.0125277.s005.pdf]

S1 Fig.

Inhibitory effects of the combination of siRNAs TMPRSS2-ERG III and IV on oncogene and oncoprotein expressions

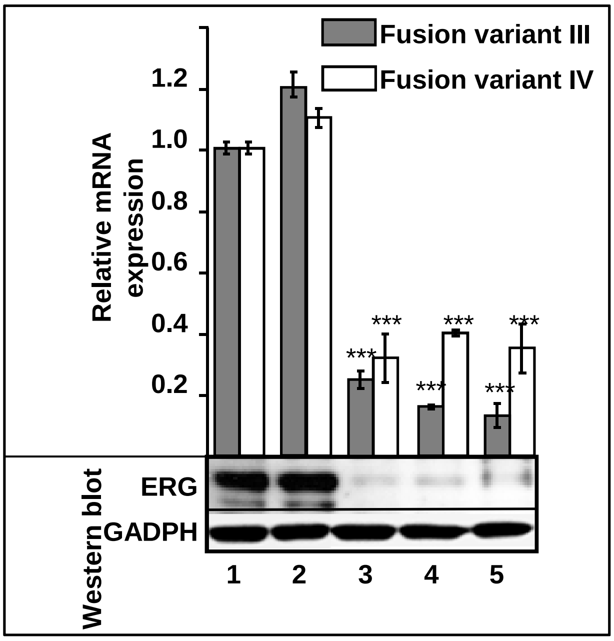

1 = Non-treated cells
2 = siRNA Control
3 = siRNA TMPRSS2-ERG III
4 = siRNA TMPRSS2-ERG IV
5 = siRNA TMPRSS2-ERG III + IV

VCaP cells were transfected for 72h with siRNAs alone (TMPRSS2-ERG III, IV and Control) or in combination (TMPRSS2-ERG III + IV) at 50nM concentration. For RT-qPCR analysis, cells were harvested, mRNA extracted and RT-qPCR was performed. Relative TMPRSS2-ERG fusion variants III and IV mRNA levels were analysed then compared to non-treated cells and results are normalised to GAPDH mRNA expression. Bars represent the mean ± SD of three independent experiments. Using Kruskal & Wallis test followed by Tukey and Dunnet tests, a statistical difference was observed between treatments compared to non-treated cells : ***=p<0.001. For Western blot analysis, ERG protein level in VCaP cells were analysed by Western blot after 72h of treatment. GAPDH was used as loading control. The figure shows one representative of the three independent experiments.
