# Supplementary material for: Antineoplastic Effects of siRNA against TMPRSS2-ERG Junction Oncogene in Prostate Cancer
Source: PLoS One. 2015 May 1;10(5):e0125277. doi: 10.1371/journal.pone.0125277 (PMC4416711; doi:10.1371/journal.pone.0125277)
Supplement: S2 Fig — VCaP cells were transfected for 72h with siRNAs alone (TMPRSS2-ERG III, IV or Control) or in combination (TMPRSS2-ERG III and IV) at 50 nM concentration and the MTT viability assay was performed. The number of viable cells was measured and compared to non-treated cells incubated with transfecting agent only (100% cell viability). Results are the mean ± SD of two independent experiments containing 8 replicates for each condition. Statistical analysis (Kruskal & Wallis followed by Tukey test) was performed to assess the difference between treatments compared to non-treated cells. ** = p<0.01, *** = p<0.001. (PDF) [file pone.0125277.s006.pdf]

S2 Fig.

Inhibitory effects of the combination of siRNAs TMPRSS2-ERG III and IV on cell viability.

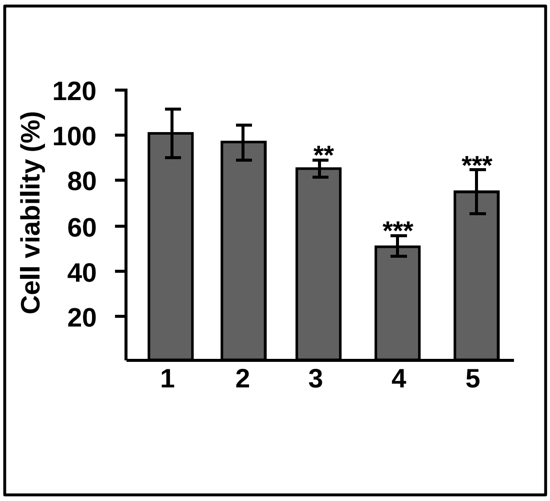

1 = Non-treated cells
2 = siRNA Control
3 = siRNA TMPRSS2-ERG III
4 = siRNA TMPRSS2-ERG IV
5 = siRNA TMPRSS2-ERG III + IV

VCaP cells were transfected for 72h with siRNAs alone (TMPRSS2-ERG III, IV and Control) or in combination (TMPRSS2-ERG III + IV) at 50nM concentration and the MTT viability assay was performed. The number of viable cells was measured and compared to non-treated cells incubated with transfecting agent only (100% cell viability). Results are the mean ± SD of 2 independent experiments containing 8 replicates for each condition. Statistical analysis (Kruskal & Wallis followed by Tukey and Dunnet test) was performed to assess the difference between treatments compared to non-treated cells. **=p<0.01; ***=p<0.001.
