# Supplementary figures and images for: Antineoplastic Effects of siRNA against TMPRSS2-ERG Junction Oncogene in Prostate Cancer
Source: PLoS One. 2015 May 1;10(5):e0125277. doi: 10.1371/journal.pone.0125277 (PMC4416711; doi:10.1371/journal.pone.0125277)

**S3 Fig.**

Effects of siRNA TMPRSS2-ERG III and IV on cell death pathways by flow cytometry

**72h**

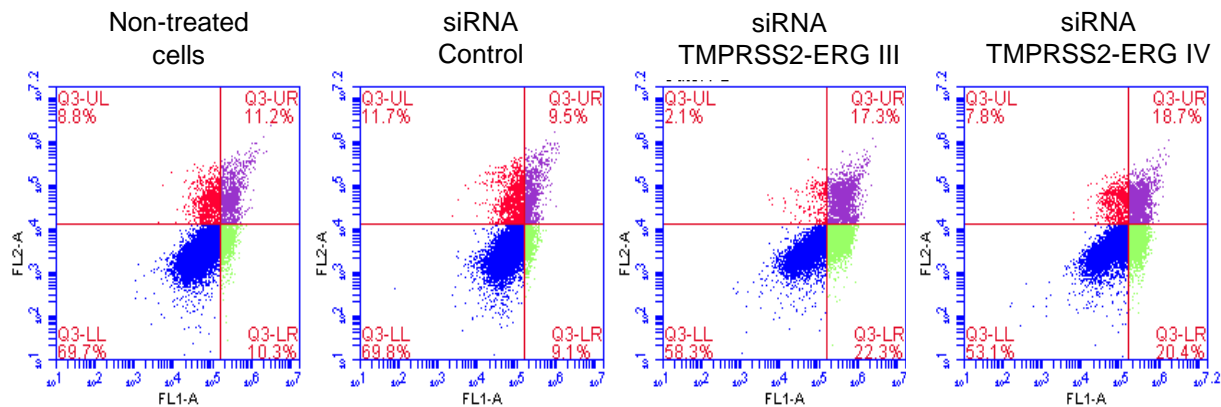

**96h**

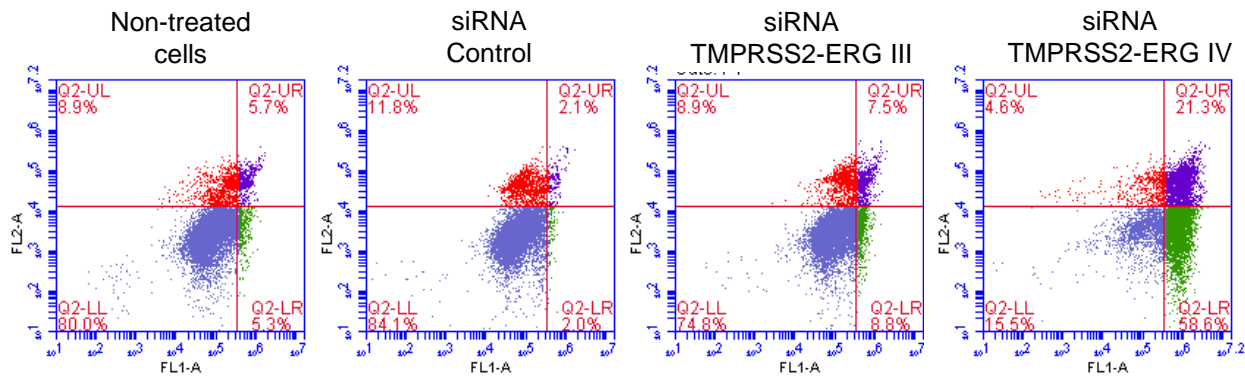

Supplement: S3 Fig — (PDF) [file pone.0125277.s007.pdf]
