# Supplementary material for: Antineoplastic Effects of siRNA against TMPRSS2-ERG Junction Oncogene in Prostate Cancer
Source: PLoS One. 2015 May 1;10(5):e0125277. doi: 10.1371/journal.pone.0125277 (PMC4416711; doi:10.1371/journal.pone.0125277)
Supplement: S4 Fig — The intracellular localisation of FAM-labelled nanoparticles was assessed by using fluorescence microscope. VCaP cells were treated with 50 nM of NPs FAM-labelled siRNA TMPRSS2-ERG IV-SQ in the presence or absence of Lipofectamine RNAiMAX and incubated at 37°C for 24 h. Cell nuclei were stained with DAPI (blue) and FAM-labelled siRNA TMPRSS2-ERG tumour (green) were observed with fluorescence microscope at 20X. Lower panel: inhibition of TMPRSS2-ERG oncogene and oncoprotein by NPs. VCaP cells were transfected for 48h with FAM-labelled siRNA TMPRSS2-ERG IV-SQ NPs in the presence or absence of transfecting agent Lipofectamine RNAiMAX (TA). Cells were then harvested and relative mRNA and ERG protein levels were analysed by RT-qPCR and Western blot respectively. Treatments correspond to: 1. Non-treated cells, 2. siRNA TMPRSS2-ERG IV-SQ NPs in the absence of transfecting agent, 3. siRNA TMPRSS2-ERG IV-SQ NPs in the presence of transfecting agent, 4. siRNA TMPRSS2-ERG IV in the presence of transfecting agent. (PDF) [file pone.0125277.s008.pdf]

S4 Fig. Uptake of Nanoparticles in VCaP cell line.

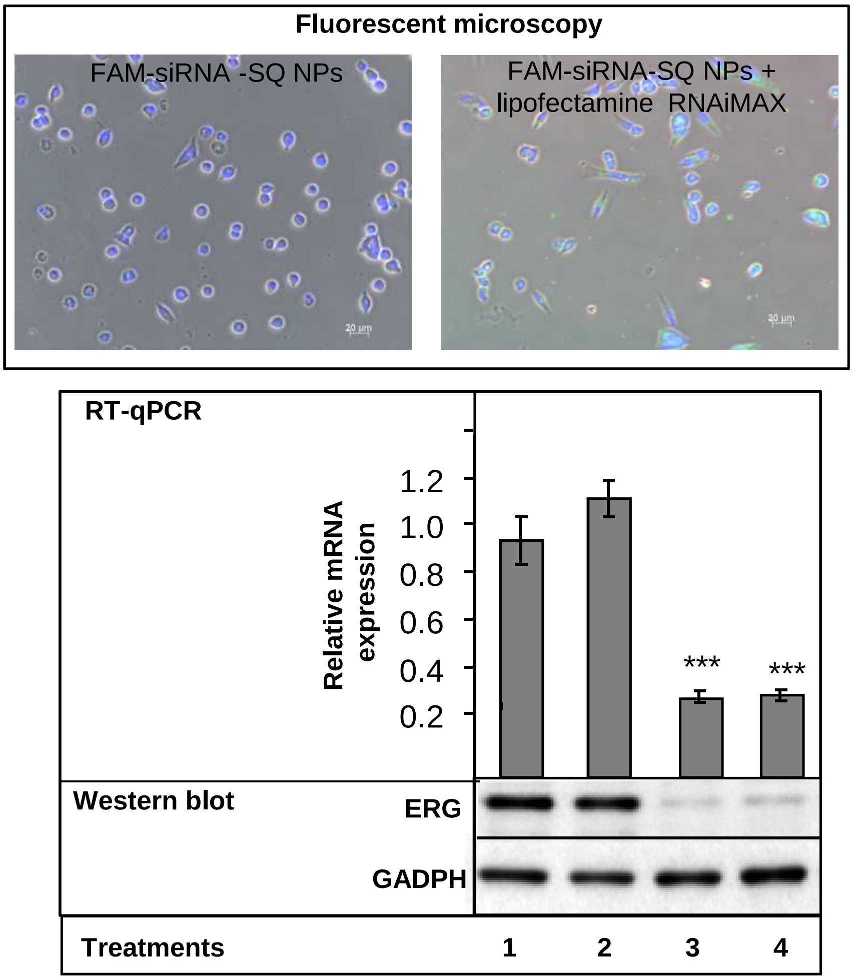

Upper panel: The intracellular localisation of FAM-labelled nanoparticles was assessed by using fluorescence microscope. VCaP cells were treated with 50 nM of NPs FAM-labelled siRNA TMPRSS2-ERG IV-SQ NPs in the presence or absence of Lipofectamine® RNAiMAX and incubated at 37℃ for 24 h. Cell nuclei were stained with DAPI **(**blue) and FAM-labelled siRNA TMPRSS2-ERG tumour cells (green) were observed with fluorescence microscope at 20X.

Lower panel: VCaP cells were transfected for 48h with FAM-labelled siRNA TMPRSS2-ERG IV-SQ NPs in the presence or absence of transfecting agent Lipofectamine® RNAiMAX (TA). Cells were then harvested and relative mRNA was analysed by RT-qPCR and ERG protein levels by Western blot. Treatments correspond to: 1. Non-treated cells, 2. siRNA TMPRSS2-ERG IV-SQ NPs in absence of the transfecting agent, 3. siRNA TMPRSS2-ERG IV-SQ NPs in presence of the transfecting agent, 4. siRNA TMPRSS2-ERG IV in presence of the transfecting agent.
